# Face masks considerably reduce COVID-19 cases in Germany

**DOI:** 10.1073/pnas.2015954117

**Published:** 2020-12-03

**Authors:** Timo Mitze, Reinhold Kosfeld, Johannes Rode, Klaus Wälde

**Affiliations:** ^a^Department of Business and Economics, University of Southern Denmark, 5230 Odense, Denmark;; ^b^Institute of Economics, Universität Kassel, 34127 Kassel, Germany;; ^c^Department of Law and Economics, Technische Universität Darmstadt, 64289 Darmstadt, Germany;; ^d^Gutenberg School of Management and Economics, Johannes-Gutenberg-Universität Mainz, D-55131 Mainz, Germany;; ^e^IZA Institute of Labor Economics, D-53113 Bonn, Germany

**Keywords:** COVID-19, public health measures, face masks, synthetic control method

## Abstract

We use the synthetic control method to analyze the effect of face masks on the spread of COVID-19 in Germany. Our identification approach exploits regional variation in the point in time when wearing of face masks became mandatory in public transport and shops. Depending on the region we consider, we find that face masks reduced the number of newly registered severe acute respiratory syndrome coronavirus 2 infections between 15% and 75% over a period of 20 days after their mandatory introduction. Assessing the credibility of the various estimates, we conclude that face masks reduce the daily growth rate of reported infections by around 47%.

## Significance

Mitigating the spread of COVID-19 is the objective of most governments. It is of utmost importance to understand how effective various public health measures are. We study the effectiveness of face masks. We employ public regional data about reported severe acute respiratory syndrome coronavirus 2 infections for Germany. As face masks became mandatory at different points in time across German regions, we can compare the rise in infections in regions with masks and regions without masks. Weighing various estimates, we conclude that 20 d after becoming mandatory face masks have reduced the number of new infections by around 45%. As economic costs are close to zero compared to other public health measures, masks seem to be a cost-effective means to combat COVID-19.

Many countries have experimented with several public health measures to mitigate the spread of COVID-19. One particular measure that has been introduced are face masks. It is of obvious interest to understand the contribution made by such a measure in reducing infections.

The effect of face masks on the spread of infections has been studied for a long time. The usefulness of facial protection for clinical personnel is beyond dispute, even though there are many questions left open (1). There is also evidence that face masks helped in mitigating the spread of earlier epidemics such as SARS 2003 (severe acute respiratory syndrome 2003) or influenza (see *SI Appendix*, section E for a brief literature survey). The impact of face masks worn in public on the spread of COVID-19 has yet to be systematically analyzed. This is the objective of this paper.

There is a general perception in Germany that the mandatory use of face masks in public reduces COVID-19 incidences considerably. This perception stems mainly from the city of Jena. After face masks became mandatory between 1 April and 10 April 2020 the number of new infections fell almost to zero. Jena is not the only region in Germany, however, that introduced face masks. Six further regions made masks compulsory before the introduction at the federal state level. Eventually, face masks became mandatory in all federal states between 20 April and 29 April 2020 (see *SI Appendix*, section A for background).

We quantify the effectiveness of face masks by employing the synthetic control method (SCM; refs. 2–5). Our identification approach exploits this regional variation in the point in time when face masks became mandatory. We use data for 401 regions in Germany (municipal districts) to estimate the effect of this particular policy intervention on the development of registered infections with COVID-19. We consider the timing of mandatory face covering as an exogenous event to the local population: Masks were imposed by local authorities and were not the outcome of some process in which the population was involved. We compare the COVID-19 development in various regions to their synthetic counterparts. The latter are constructed as weighted averages of control regions that are structurally similar to treated regions. Structural dimensions considered include prior COVID-19 cases, the demographic composition, and the local health care system.

A detailed analysis of the timing of all public health measures in the regions we study guarantees that we correctly attribute our findings to face masks (and not erroneously to other public health measures). We also employ a standard SIR (susceptible–infected–removed) model and undertake an analysis of the distribution of the lag between infection and reporting date. This allows us to provide a precise interpretation of our empirical effectiveness measure and to pin down the point in time when the effects of face masks should be visible in the data.

We find statistically significant and sizeable support for the general perception that the public wearing of face masks in Jena strongly reduced the number of incidences. We obtain a synthetic control group that closely follows the COVID-19 trend before the introduction of mandatory masks in Jena. The difference between Jena and this group becomes significant thereafter. Our findings indicate that the early introduction of face masks in Jena has resulted in a drop in newly registered COVID-19 cases of around 75% after 20 d. Put simply, if the control region observes 100 new infections over a period of 20 d, the mask region observes only 25 cases. This drop is greatest, by more than 90%, for the age group 60 y and above. Our results are robust to different sensitivity checks, among which are placebo-in-space and placebo-in-time analyses.

As a means to verify the generalizability of our findings for Jena, we move from a single- to a multiple-treatment approach and estimate average treatment effects of introducing face masks on the spread of COVID-19 for all regions that introduced masks by 22 April (∼8% of all German regions). Although the estimated average treatment effect is smaller compared to the one found for Jena, it is still statistically significant and sufficiently large to support our point that wearing face masks is an effective and cost-efficient measure for fighting COVID-19. When we summarize all of our findings in one single measure (*SI Appendix*, section D.2), we conclude that the daily growth rate of COVID-19 cases in the treatment group falls by around 47% due to mandatory mask-wearing relative to the synthetic control group.*

Our findings can be aligned with earlier evidence on face masks, public health measures, and the epidemic spread of COVID-19, although consolidated scientific knowledge is limited (*SI Appendix*, section E). While there is a growing consensus from clinical studies that face masks significantly reduce the transmission risk of severe acute respiratory syndrome coronavirus 2 (SARS-CoV-2) and COVID-19 (7, 10), nonclinical evidence on the effectiveness of face masks is still largely missing. Ref. 11 surveys evidence on the population impacts of a widespread community mask use and stresses that “no randomized control trials on the use of masks … has been published.” The study which is “the most relevant paper” for ref. 11 is one that analyzed “exhaled breath and coughs of children and adults with acute respiratory illness” (ref. 12, p. 676), that is, used a clinical setting. Concerning the effect of masks on community transmissions, the survey needs to rely on pre–COVID-19 studies.

Ref. 13 is among the first to estimate the population impact of face masks on SARS-CoV-2 infections.^†^ The authors track the development of COVID-19 in three pandemic epicenters, Wuhan, Italy, and New York City, between 23 January and 9 May 2020 and find sizable mitigating effects of face masks on epidemic spread. While their study offers important insights into the population effects of face masks, a methodical limitation is that estimates are only carried out in a “before–after” manner with no use of a strict control group approach. This may limit the causal interpretation of results. We therefore follow the spirit of ref. 4 and provide causal evidence identifying the population impact of mandatory face masks on the spread of COVID-19.

## Results: The Effects of Face Masks on the Spread of COVID-19

All results are obtained by applying the synthetic control method. It is described briefly in *Method and Data* and in more detail in *SI Appendix*, section B.

### Results for Jena.

Face masks became mandatory in Jena in three steps between 1 and 10 April. The most important measure (in the sense of having the largest impact measured in terms of social contacts) requires face masks in public transports and shops and entered into force on 6 April (see *SI Appendix*, section A for detailed information). We therefore center our discussion on this date.

**Fig. 1.**
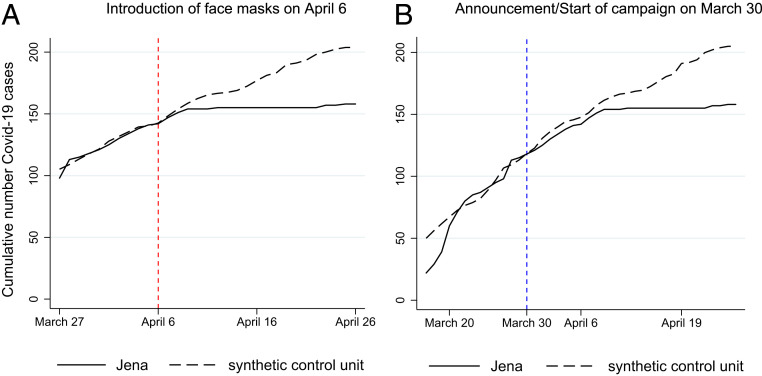
Treatment effects of the mandatory introduction of face masks (6 April) and of its announcement (30 March) in Jena. The figure shows the development of the cumulative number of COVID-19 cases in Jena (treated region, black solid line) compared to a synthetic control group (gray dashed line) over time. (Details on the construction of the synthetic control group and SCM estimation are given in *Method and Data*.) Both panels distinguish between a pretreatment and treatment period. In *A*, the treatment period starts on 6 April when face masks became mandatory in public transport and shops. The start of the treatment period is indicated by the dashed vertical line (red). In *B*, the treatment period is set to begin on 30 March as starting date of the local campaign in Jena to wear face masks in public. The start of the treatment period is indicated by the dashed vertical line (blue). The panels show that the conclusion is independent of the starting dates: Face masks strongly reduced the number of COVID-19 cases in Jena.

Fig. 1*A* shows the SCM results for the introduction of face masks in Jena on 6 April. The visual inspection of the development of cumulative COVID-19 cases shows that the trend development of the synthetic control group is very similar to Jena before the treatment, indicating a good fit.^‡^ The difference in the cumulated registered COVID-19 cases between Jena and its corresponding synthetic control group after the start of the treatment on 6 April can be interpreted as the treatment effect on the treated [see *SI Appendix*, section C.3 for (post)estimation details].

Fig. 1*A* clearly shows a gradually widening gap in the cumulative number of COVID-19 cases between Jena and its synthetic control group. The size of the effect 20 d after the start of the treatment (6 April) amounts to a decrease in the number of cumulative COVID-19 cases of 23%, which corresponds to a drop in newly registered cases of roughly 75%. Expressed differently, the daily growth rate of the number of infections decreases by 1.28 percentage points per day (see *SI Appendix*, section D.2 for computational details and an overview of all measures). If we look at the estimated differences by age groups, *SI Appendix*, Fig. S7 indicates that the largest effects occur for individuals above 60 y of age. Here the reduction in cumulative cases even exceeds 50%, which corresponds to a drop in newly registered SARS-CoV-2 infections by more than 90%. The significant drop can be explained by the introduction of face masks in elderly and nursing homes, which had already started on 1 April. For the other two age groups the decrease in the number of cumulative cases lies between 10% and 20%.

If we consider a median time lag of 10.5 d from infection until registration (see *SI Appendix*, section A.3), the occurrence of a gradually widening gap between Jena and its synthetic control in the first week after the introduction of mandatory face masks seems fast. One might conjecture that an announcement effect played a role. As shown in *SI Appendix*, section C.6.1, online searches for (purchasing) face masks peaked on 22 April, when it was announced that face masks would become compulsory in all German federal states.^§^ Another peak in online searches, almost as large (70% of the peak of 22 April), appeared on 31 March. This marks the date of the regulation making masks compulsory between 1 and 10 April in Jena. The regulation was accompanied by a campaign “*Jena zeigt Maske*,” communicating the necessity to wear face masks in public, that started on 30 March.^#^

Fig. 1*B* plots the estimated effect size when we define the start of the treatment period by the start of the campaign on 30 March. The visual inspection of the difference between Jena and its synthetic control group points to the presence of a small anticipation effect. Yet, the gap to the synthetic control significantly widens only ∼10 to 12 d after the announcement and then grows considerably over time. As this temporal transmission channel appears plausible given a median time lag between infection and registration of almost equal length, we take this as first evidence for a face mask effect in the reduction of SARS-CoV-2 infections.

### Robustness Checks.

Obviously, the estimated difference in COVID-19 development in Jena vis-à-vis the synthetic Jena is only convincing if 1) the requirements of the SCM are fulfilled and 2) potentially concurrent policies other than masks can be ruled out. The role of the first set of robustness checks consists of understanding the sensitivity to the length of the preintervention period (*Cross-Validation Tests*) and to the composition of the control pool (*Changing the Donor Pool*).^ǁ^ In a second step, we rule out unobserved macro effects shared by many regions (*Placebo-in-Space Tests*) and test for anticipation effects potentially caused by other public health measures (*Placebo-in-Time Tests*). The second step also comprises difference-in-difference estimation as a further test for latent concurrent policies that go beyond observable policies (displayed in *SI Appendix*, Fig. S2).

#### Cross-validation tests.

We study the sensitivity of our estimates with respect to the length of the training and validation period before the start of the treatment. We accordingly alter the imposed lag structure for predictors that have a time dimension, that is, the number of cumulative and newly registered COVID-19 cases. The set of time-constant predictors is kept unchanged by this test. As shown in *SI Appendix*, section C.7, we do not find a systematic estimation bias of our baseline SCM specification compared to alternative ones with longer lag structures (up to 7 d) and accordingly shorter trainings periods. Given that regional COVID-19 cases developed very dynamically and nonlinearly in the pretreatment period, this is an important finding in terms of the robustness of our results.

We also test for the sensitivity of the estimated treatment effects to changes in the set of time-constant predictors. We do so by sequentially excluding individual variables from the set of predictors. As shown in *SI Appendix*, section C.7, the estimated trajectories for the respective synthetic control groups follow a very similar trend. All of them identify a reduction in the number of cumulative COVID-19 cases in Jena vis-à-vis the synthetic Jena that widens over time. We conclude from these results that using the full set of predictors is the most reasonable approach.

#### Changing the donor pool.

This may be equally important as our baseline specification includes the region of Heinsberg in the donor pool used to construct the synthetic Jena (with a weight of 4.6%; compare *SI Appendix*, Table S5). As Heinsberg is one of the German regions that was significantly affected by the COVID-19 pandemic during the Carnival season, one may expect that this leads to an overestimation of the effects of face masks. Accordingly, *SI Appendix*, section C.8 presents estimates for alternative donor pools. Again, we do not find evidence for a significant bias in our baseline specification. By tendency, the treatment effect becomes larger, particularly if we compare Jena only to other regions in Thuringia (to rule out macroregional trends) and to a subsample of larger cities (*kreisfreie Städte*). The latter comparison reduces the degree of latent regional heterogeneity, for instance, with regard to social interactions. Both subsamples exclude Heinsberg. We also run SCM for subsamples excluding Thuringia (to rule out spatial spillover effects) and for East and West German regions only (again to test for specific macro regional trends). Generally, these sensitivity tests underline the robustness of the estimated treatment effect for Jena.

#### Placebo-in-space tests.

These tests check whether other cities that did not introduce face masks on 6 April have nonetheless experienced a similar decline in the number of registered COVID-19 cases. If this had been the case, the treatment effect might have been driven by other latent factors rather than by face masks. Such latent factors may, for instance, be related to the macroregional dynamics of COVID-19 in Germany. Therefore, *SI Appendix*, section C.9 reports pseudo-treatment effects for similarly sized cities in the federal state of Thuringia assuming that they had introduced face masks on 6 April—although, in fact, they did not. As the figure illustrates, these cities show either a significantly higher or a similar development of registered COVID-19 cases compared to their synthetic controls. This result provides further empirical support for a relevant effect in the case of Jena.

**Fig. 2.**
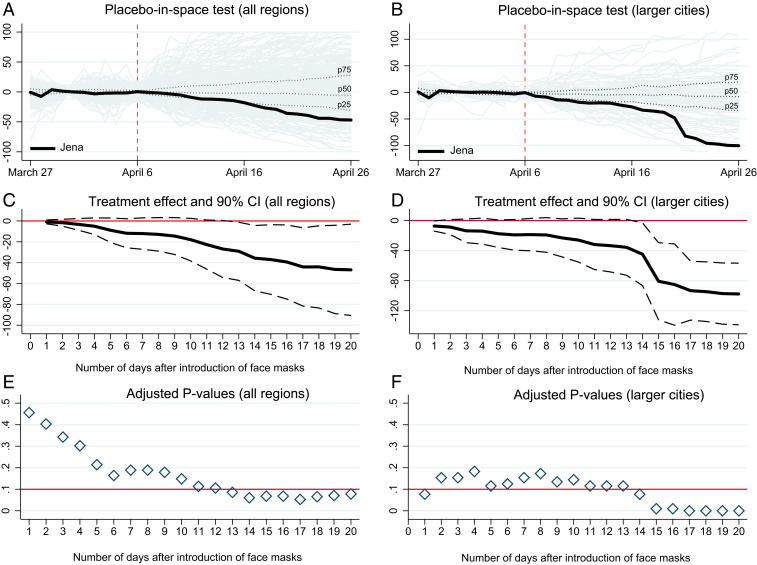
Comprehensive placebo-in-space tests for the effect of face masks on COVID-19 cases. The figure compares the difference in the cumulative number of COVID‐19 cases between Jena and its synthetic control group with the differences between all other regions in the donor pool and their respective controls over time. Differences have been calculated for the treatment period after 6 April when Jena de facto introduced mandatory face masks. For all other regions, the reported differences in the treatment period constitute pseudo‐treatment effects since these regions had not introduced face masks back then. In *A*, the black solid line measures the treatment effect for Jena. The solid gray lines measure the pseudo‐treatment effects for all other German regions in the donor pool (see *Method and Data* for details on the specification of the donor pool). Plots are shown for donor regions with an acceptable pretreatment root-mean-square prediction error (RMSPE), that is, less than 10 times the RMSPE of Jena; dotted lines indicate the median (p50), 25th percentile (p25), and 75th percentile (p75) of pseudo-treatment effects. In *B*, the donor pool is reduced to comprise only larger cities (*kreisfreie Städte*). *C* and *D* also plot the treatment effect for Jena and 90% confidence intervals (gray dashed lines) for the full sample of regions and the subsample of larger cities, respectively. Confidence intervals are constructed on the basis of pseudo *P* values as shown in *E* and *F* for the first 20 d after the introduction of face masks in Jena. These *P* values are adjusted for the pretreatment match quality (see *Method and Data* for details). The red horizontal line in *E* and *F* indicates a threshold *P* value of 0.1.

As a more comprehensive test, we run placebo-in-space tests for all other regions that did not introduce face masks on 6 April or closely afterward. Again, we estimate the same model on each untreated region, assuming it was treated at the same time as Jena. The empirical results in Fig. 2 indicate that the reduction in the reported number of COVID-19 cases in Jena clearly exceeds the trend in most other regions—both for the overall sample in Fig. 2*A* and the subsample of large cities (*kreisfreie Städte*) in Fig. 2*B*.

One advantage of these tests is that they allow us to conduct inference on the significance of the estimated treatment effects for Jena. Accordingly, Fig. 2 *C* and *D* visualize the estimated treatment effects together with 90% confidence intervals. Intervals have been calculated on the basis of (one-sided) *P* values (pretreatment match quality adjusted) reported in Fig. 2 *E* and *F*.** The latter indicate the probability that the reduction in the number of COVID-19 cases was observed by chance given the distribution of pseudo-treatment effects in the other German regions (see ref. 18). In both panels, the reported confidence intervals and underlying *P* values indicate that the reduction in the number of COVID-19 cases was not a random event. In larger cities (Fig. 2*D*), the reduction due to the introduction of face masks is clearly visible 2 wk after the start of the treatment. Again, this timing is in line with our above argument that a sufficiently long incubation time and testing lags need to be considered in the evaluation of treatment effects.^††^

#### Placebo-in-time tests.

As for the case of placebo-in-space tests, it is important for the validity of results that we do not observe significant treatment effects for Jena prior to the introduction of face masks on 6 April or its announcement on 30 March. To rule out such anticipation effects, we have systematically reviewed all general decrees published by the local administration in Jena. Of particular interest are those decrees that significantly differ with respect to their timing from those at the federal state level in Thuringia.

Looking at *SI Appendix*, Fig. S2, Jena and Thuringia passed at least 40 public health measures before the end of April 2020. Jena implemented 27 of these 40 either earlier than Thuringia or on its own. Examples of earlier implementation include the closing of bars, cafés, and restaurants or quarantine rules for travelers returning home. Relevant regions included foreign countries but also other German federal states, among which were Bavaria, Baden-Wurttemberg, and North-Rhine Westphalia. Measures imposed by Jena only include the complete closing of hotels (in contrast to closing of hotels for tourism only in Thuringia) and a curfew (which lasted for only 2 wk, though).

**Fig. 3.**
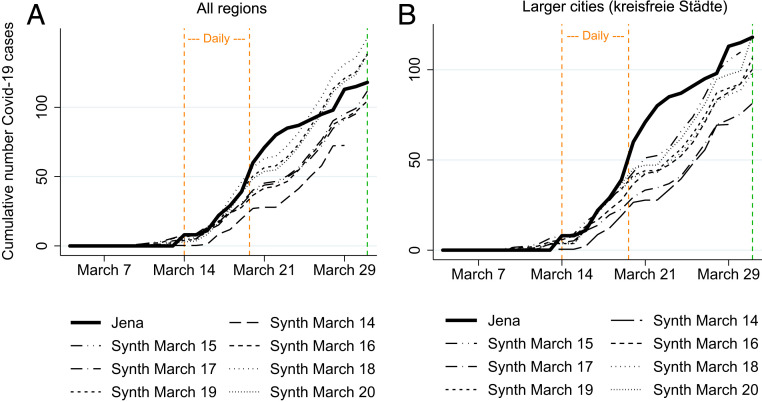
Placebo-in-time tests for (pseudo) treatment effects in the period 14 to 20 March. The figure shows the empirical development of the cumulative number of COVID-19 cases in Jena (treated region, black solid line) and the estimated development in different synthetic control groups over time. The key difference between these synthetic control groups lies in the starting point of the (pseudo) treatment period. The starting point varies on a daily basis between 14 and 20 March. In *A*, the full sample of German regions is used as the donor pool for the construction of synthetic control groups (see *Method and Data* for details on the specification of the donor pool). Vertical dashed lines (orange) indicate the time corridor in which the respective (pseudo) treatment periods start. In *B*, the donor pool is reduced to comprise only larger cities (*kreisfreie Städte*). Again, vertical dashed lines (orange) indicate the time corridor in which the respective (pseudo) treatment periods start.

As these major health decrees were accompanied by smaller ones on an almost daily basis until 20 March, we run a series of SCM estimations using each day between 14 and 20 March as a (pseudo)treatment period.^‡‡^ The results for the full donor pool including all other German regions and the subsample of larger cities are shown in Fig. 3 *A* and *B*. Results are reported until 30 March when the mandatory introduction of face masks was announced.

To take an example, consider the graph with the long dashes in Fig. 3*A* denoted “Synth March 14.” It shows the cumulative number of COVID-19 cases for a synthetic control group for Jena extracted from the full donor pool under the assumption that some treatment had started on 14 March. Similarly, “Synth March 15” (and so on) show the evolution of COVID-19 cases for a starting date of 15 March. Hence, each SCM estimation starts at a different point in time.

The visual inspection of the relative development of COVID-19 cases in Jena vis-à-vis its synthetic Jena does not indicate a clear treatment effect in terms of reducing COVID-19 cases prior to 1 April. The results are particularly clear-cut for the sample of larger cities in Fig. 3*B*, indicating that earlier public health measures alone did not significantly suppress the number of COVID-19 cases in Jena in the first 2 wk after their introduction.

#### Difference-in-difference tests.

The reported trajectories of synthetic Jena in Fig. 3*A* leave us with some degree of ambiguity, though. To explicitly test for a potential trend reversal in the development of COVID-19 cases prior to the introduction of face masks, we further run an alternative robustness test on the basis of incremental difference-in-difference (DiD) estimation. The DiD estimator is particularly well-suited to estimate dynamic treatment effects in the context of limited information about the exact length of transmission lags before individual policy interventions show measurable effects (a detailed description is given in *SI Appendix*, section F). As the results clearly show, treatment effects from public health measures in Jena in terms of a reduction in COVID-19 cases only become statistically significant roughly 2 wk after the introduction of face masks on 6 April. If we resort to the estimated incubation and reporting lag as shown in *SI Appendix*, section A.3, this result supports our main SCM findings that the relative reduction in the cumulative number of COVID-19 cases is mainly attributable to the timing of introducing face masks. The incremental DiD results also support our main SCM findings in terms of the magnitude of the treatment effect.

### Results for Other Regions.

Jena may be a unique case. We therefore also study treatment effects for other individual regions that introduced face masks earlier than other regions. Further single-unit treatment analyses are shown in *SI Appendix*, section D.1. SCM estimation for multiple treated units can be undertaken as not only did some individual regions introduce face masks earlier than their federal states (see Fig. 5, below the time line) but also some federal states (Saxony and Saxony-Anhalt) before the remaining German federal states (*SI Appendix*, Fig. S1). To ensure a sufficiently long treatment period, we consider all regions as treated which introduced face masks on or before 22 April. We estimate average treatment effects in a multiple treatment SCM approach for 1) all of these regions and 2) a subset consisting of larger cities (*kreisfreie Städte*) only. In the former case we have a total of 32 treated units, and in the latter there are 8 treated units. The donor pool of control regions (all regions in Germany and only larger cities, respectively) is specified such that the minimum time lag in the introduction of face masks between treated and control regions ranges between 5 and 13 d.

**Fig. 4.**
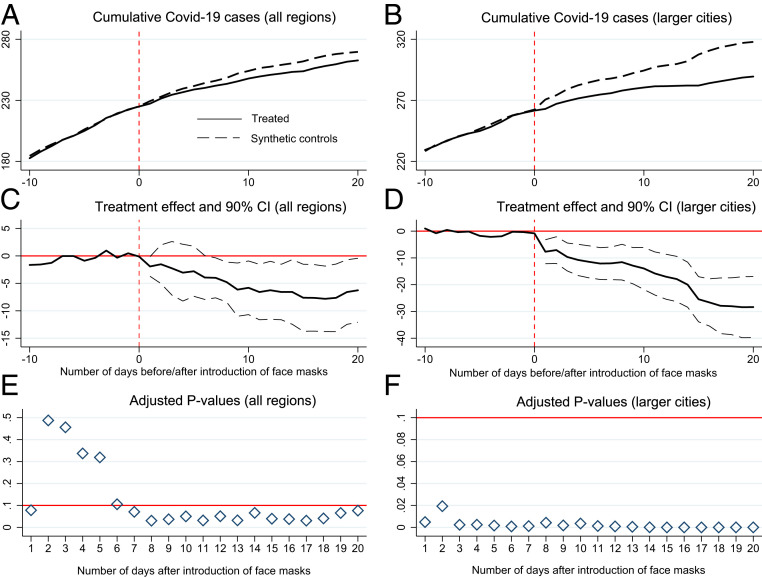
Average treatment effects for the introduction of face masks with multiple treated units. The figure shows the average development of the cumulative number of COVID-19 cases for treated units (defined as all regions that introduced face masks on or before 22 April), for their synthetic control groups and estimated treatment effects. *A* plots the average number of cumulative COVID-19 cases in treated regions (black solid line) and synthetic controls (black dashed line) for the full sample of German regions in the donor pool (see *Method and Data* for details on the specification of the donor pool). The horizontal axis plots the number of days before and after the start of the treatment (mandatory introduction of face masks). The vertical dashed line (red color) indicates the start of the treatment period, which is allowed to vary by treated regions. In *B*, the number of treated and the donor pool for control regions is limited to larger cities (*kreisfreie Städte*). *C* and *D* plot the estimated average treatment effects, that is, average reduction in the cumulative number of COVID-19 cases (black solid lines) over time joint with 90% confidence intervals (gray dashed lines) for the two samples. Confidence intervals are constructed on the basis of pseudo *P* values as shown in *E* and *F* for the first 20 d after the start of the treatment. These (one-sided) *P* values are adjusted for the pretreatment match quality (see *Method and Data* for details). Inference has been conducted on the basis of a randomly drawn sample of 1 million placebo averages. The red horizontal line in *E* and *F* indicates a threshold *P* value of 0.1.

The results, visible in Fig. 4, point to a significant face mask effect in the reduction of SARS-CoV-2 infections over a period of 20 d after the introduction. The temporal evolution of the average number of cumulative COVID-19 cases for treated regions and their corresponding synthetic control groups are shown in Fig. 4 *A* and *B*, respectively. The reported 90% confidence intervals in Fig. 4 *C* and *D* calculated on the basis of adjusted *P* values shown in Fig. 4 *E* and *F* indicate that the estimated treatment effects are not random for both samples. While treatment effects of face masks turn significant after roughly 1 wk for the overall sample, the emergence of a reduction in the subsample of larger cities is fast and points to early anticipation effects of face masks in urban areas, particularly during the period when local economies were gradually reopened after 20 April.

Importantly, however, the trend development for larger cities as shown in Fig. 4 not only indicates a drop in the number of newly registered COVID-19 cases around the immediate timing of the introduction of face masks but also points to the presence of dynamic treatment effects as the average gap between treated regions and their synthetic control groups widens over time. This hints at the role played by mandatory face masks in avoiding a new wave of new infections once the economy and labor market is reopened. As Fig. 4*B* highlights, such an avoidance effect may be particularly important in larger cities with higher population density and accordingly higher intensity of social interaction^§§^

Taken together, over a period of 20 d, we observe an average reduction of 28.4 cases between treated and control regions in the context of urban areas. Relative to the average number of cumulative COVID-19 cases on 11 May in control regions (317.9), this amounts to a reduction of 8.9% in the cumulative number of COVID-19 cases and a reduction of 51.2% in newly registered cases. The difference in the daily growth rate of the number of infections correspondingly amounts to 0.46 percentage points. For the full sample, this difference is estimated to be 0.13 percentage points (see *SI Appendix*, section D.2 for an overview of all measures and *Method and Data* for theoretical background). This smaller magnitude in the latter sample including all municipal districts has to be evaluated against the background of a considerable degree of structural heterogeneity, for instance, related to the composition of the local population but also the local COVID-19 spread. We argue that the latter should thus be interpreted as a lower bound for the true treatment effects.

## Discussion

We set out by analyzing the effect of face masks on the spread of COVID-19 for a comparative case study of the city of Jena. Our quasi-experimental control group approach using SCM shows that the introduction of face masks on 6 April reduced the number of newly registered COVID-19 cases over the next 20 d by 75% relative to the synthetic control group. Comparing the daily growth rate in the synthetic control group with the observed daily growth rate in Jena, the latter shrinks by around 70% due to the introduction of face masks. This is a sizeable effect. The introduction of mandatory face masks and the associated signal to the local population to take the risk of person-to-person transmissions seriously apparently helped considerably in reducing the spread of COVID-19. Looking at average treatment effects for all other regions puts this result in some perspective. The reduction in the daily growth rate of infections amounts to 14% only. By contrast, when we focus on larger cities, we find a reduction in the daily growth rate of infections by roughly 47%.

What would we reply if we were asked what the effect of introducing face masks would have been if they had been made mandatory all over Germany? The answer depends, first, on which of the percentage measures we found above is the most convincing and, second, on the point in time when face masks are made compulsory. The second aspect is definitely not only of academic interest but would play a major role in the case of a second wave.^##^

We believe that the reduction in the daily growth rates of infections between 47% and 70% is our best estimate of the effects of face masks. Arguments in favor of the high 70% stress that Jena introduced face masks before any other region did so. It announced face masks as the first region in Germany while in our posttreatment period hardly any other public health measures were introduced or eased. Hence, it provides the most clear-cut quasi-experimental setting for studying its effects. Second, as described in *Method and Data*, Jena is a fairly representative region of Germany in terms of COVID-19 cases. Third, the smaller treatment effects observed in the multiple-treatment analysis may also result from the fact that—by the time that other regions followed the example of Jena—behavioral adjustments in Germany’s population had already taken place. Wearing face masks gradually became more common and more and more people started to adopt their usage even when it was not yet required. The results for the subsample of larger cities are, however, quantitatively similar to Jena.

Arguments for the lower 47% state that the stronger impact of face masks on the infectious in Jena may thereby partly be driven by a Hawthorn effect. The population in this pioneer region might have reacted very strongly to the mandatory introduction of face masks by taking the other imposed public health measures and hygiene rules (washing hands, limiting interactions, staying at home more, etc.) more seriously.

Concerning the point in time (or better, the point in the epidemic cycle) when face masks become mandatory, all of our estimates might actually be modest. The daily growth rates in the number of infections when face masks were introduced in Jena was around 2 to 3%. These are low growth rates compared to the early days of the epidemic in Germany, where daily growth rates lay above 50% (20). One might therefore conjecture that the effects might have been even greater if masks had been introduced earlier.

This timing effect might also explain the difference between Jena estimates and lower estimates for other regions. By the time Jena introduced face masks on 6 April, the general trend in development of COVID-19 cases was still relatively dynamic across German regions. In mid-April, when other regions followed the example of Jena and introduced face masks before the general introduction at the federal state level, overall daily growth rates were already lower.

We simultaneously stress the need for further complementary analyses. First, Germany is only one specific country. Different regulations, norms (which relate to compliance), or climatic conditions might change the empirical picture for other countries. Second, we ignored the impact of the number of tests on reported infections. While we do not believe that this matters for Germany as rules for testing are homogenous across regions, this might play a bigger role for international comparisons. Third, we have ignored spatial dependencies in the epidemic diffusion of COVID-19. This might also matter. Fourth, there are various types of face masks. We cannot identify differential effects since mask regulations in German regions do not require a certain type. Finally, economic costs should be taken into account. When we compare masks with other common measures,*** the implied economic costs for community masks seem comparatively low. This applies to disposable masks and reusable nonmedical masks. Yet, the cost of information campaigns should be taken into account. While a detailed cost–benefit analysis is needed, we would expect that a comparison with other policy actions would speak in favor of face masks. [See *SI Appendix*, section E.3 for a brief introduction to the literature. We estimate that costs (of households only) for face masks amount to 1.4 to 2.5% of disposable income.]

## Method and Data

### Variation in Timing.

**Fig. 5.**
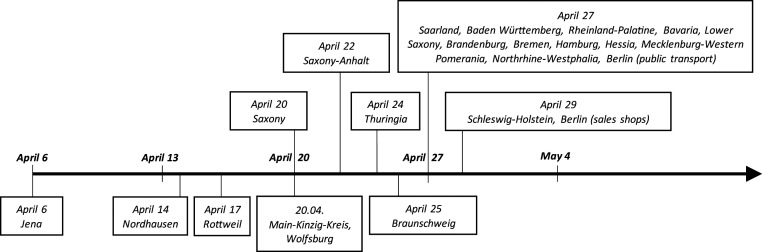
The timing of mandatory face mask wearing in German federal states (*Top*) and individual regions (*Bottom*). The figure shows the regional variation in the introduction of face masks in public transport and shops over time. While text boxes above the timeline on the horizontal axis indicate the timing when the wearing of face masks became compulsory in the respective federal states (NUTS1 level), text boxes below the timeline identify individual NUTS3 regions that have anteceded the general introduction of face masks at the federal state level. The first NUTS3 region that introduced mandatory face masks in Germany was Jena on 6 April. By 29 April face masks had become mandatory in all German regions. (See *SI Appendix*, section A.1 for more background.)

Six regions in Germany (municipal districts, equivalent to the European Union nomenclature of territorial units for statistics, NUTS, level 3 categorization) made face masks mandatory before their respective federal states. They are displayed in Fig. 5. The figure also shows differences across federal states in the timing of introducing mandatory face masks.

### Statistical Method.

We estimate treatment effects from introducing face masks by means of the SCM for single and multiple treated units. (All analyses are undertaken in Stata. The corresponding files plus data are available in *SI Appendix*.) The SCM has become increasingly popular for policy evaluations that rely on comparative case studies. The intuition of SCM estimation can briefly be described as follows (see ref. 2–5 and *SI Appendix*, section B for more background).

First, the method can be applied to policy interventions (thereafter treatment) which only target a small number of treated units (in our case, one or a few regions). Treatment effects are identified by comparing the development of outcomes in treated and control regions during the treatment period. For causal inference, the proper selection and weighting of control regions is crucial to credibly estimate what outcome would have been observed in the treated region in the absence of the treatment. To establish this counterfactual, the SCM approach constructs a synthetic control group as weighted average of regions in the donor pool of controls. Weights for individual control regions vary between 0 and 1, sum up to 1 over all control regions, and are determined on the basis of a minimum distance approach. The latter involves a set of predictor variables chosen by the researcher as to closely match the outcome of the treated region prior to the treatment (predictors used here are listed in *SI Appendix*, Table S4 and selected weights for control regions are displayed in *SI Appendix*, Table S5).

Second, statistical significance of the estimated treatment effect is based on permutation. The SCM estimates a series of placebo treatment effects for all regions in the donor pool, that is, each region in the donor pool is treated as if it had been treated. The distribution of placebo effects is then compared with the treatment effect for the treated region. If the magnitude of the latter effect is large relative to the distribution of the placebo effects, the treatment effect is considered not to be observed by chance, that is, it is deemed to be significant.

Third, the effective use of SCM relies on contextual requirements: 1) A donor pool of controls is available, that is, not all regions receive the treatment during the period of the study; 2) predictor values of the treated region are not extreme relative to those of controls, that is, the treated region lies in the convex hull of control regions; 3) data are available for a sufficiently long time horizon before and after the start of the treatment; 4) spillover effects of the treatment on controls are absent; and 5) there are no early anticipation effects. Implications of these requirements for our analysis are discussed in *SI Appendix*, section B and we apply a series of robustness tests to check if the requirements hold in our data settings (reported in *Robustness Checks*).

Finally, as we also employ DiD estimation, we briefly touch upon the relationship between these two approaches. Similar to SCM, the DiD approach estimates treatment effects by contrasting changes in outcomes between a pretreatment and treatment period for treated and nontreated (control) regions. An attractive feature of the DiD approach is its flexibility in estimating dynamic treatment effects, that is, those that build up over time potentially determined by (unobserved) interventions starting at different time periods. (This is the reason why we employed DiD above.) By contrast, DiD has its limits when only one or a few treated regions are available as heteroscedastic errors might occur (see ref. 21). DiD estimation also relies strongly on the validity of the parallel trends assumption: It requires that treated and control regions would have followed parallel trajectories over time if treatment had not occurred. Given the highly dynamic development of regional COVID-19 cases and the likely presence of macroregional trends, the validity of the common trend assumption is questionable in our data settings. For SCM estimation, we do not need to impose this assumption as the presence of common trends between treated and control regions is in itself a favorable factor for finding an appropriate counterfactual trajectory (2, 3, 22).

### Data.

We use the official German statistics on reported COVID-19 cases from the Robert Koch Institute (23). We build a balanced panel for 401 NUTS level 3 regions and 105 d spanning the period from 28 January to 11 May 2020 (42,105 observations). We use the cumulative number of registered COVID-19 cases in each district and the number of cumulative COVID-19 cases per 100,000 inhabitants as main outcome variables. We estimate overall effects for these variables together with disaggregated effects by age groups (persons aged 15 to 34 y, 35 to 59 y, and 60+ y). We also employ regional data to inter alia identify control regions. *SI Appendix*, Table S4 shows summary statistics.

Face masks are clearly not the only public health measures to mitigate the spread of COVID-19. Identification of the face mask effect therefore needs to take the timing of other public health measures into account. To this end, we built a database for all public health measures in Jena and Thuringia and for face masks in all other federal states. See *SI Appendix*, sections A.1 and A.2 for details. The database indicates that our results indeed capture the effects of face masks and not of other public health measures.

### Conceptional Background.

**Fig. 6.**
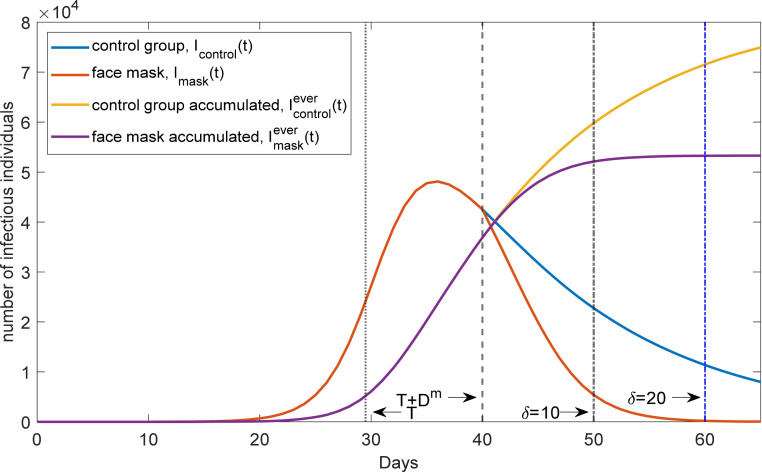
Theoretical effects of face masks on the number of infectious individuals I(t) and on the accumulated number of infectious individuals I^ever^(t). The horizontal axis plots time and the vertical axis the number of infectious individuals, both new cases (blue and red curves) and accumulated cases (yellow and purple curves). The curves show time paths following from a standard SIR model. We let an intervention take place on day *T* = 29. After a delay of D^m^ days, where D^m^ is the median of the delay between infection and reporting, the effect of the intervention is visible. Waiting 10 or 20 d then allows us to quantify the effect of the intervention.

To facilitate the interpretation of our findings, we employ a standard SIR model with three states: susceptible, infectious, and removed (see *SI Appendix*, section A.4 for more details.) Imagine we study a region where face masks are not mandatory. The time path *I*(*t*) of infections individuals in this (synthetic) control group is displayed in Fig. 6 as *I*_*control*_(t). The time path for *I*^*ever*^(t) in the control group is denoted by $I_{control}^{ever}\left(\text{t}\right)$. Now consider the introduction of mandatory face masks at *T* (set to 29.5 in Fig. 6).^†††^ Mandatory masks reduce the infection rate (via a parameter *r* in the SIR model). Given a (median) delay of *D*^*m*^ between infection and reporting to authorities (estimated at 10.5 d in *SI Appendix*, section A.3), we model this delay by effectively reducing *r* at *T + D*^*m*^. Hence, as of *T + D*^*m*^, the number of infectious individuals falls faster, see “face masks *I*_*mask*_(t),” and the number of individuals ever infected rises less quickly, as visible when looking at $I_{mask}^{ever}\left(\text{t}\right)$. Note the qualitative similarity between the yellow and purple curve here and the corresponding curves in Figs. 1*A* and 4 *A* and *B*.

Now imagine we want to quantify the effect of face masks. The model suggests that the effect of face masks can be described by the reduction in the total number of individuals ever infected. As an example, consider time *T + D*^*m*^*+δ*, that is, *δ* days after face masks became effective. The difference between the control region and the face-mask region is given by $I_{control}^{ever}\left(T+D^{m}+\delta \right)-\;I_{mask}^{ever}\left(T+D^{m}+\delta \right)$. Hence, the introduction of face masks reduced the number of COVID-19 cases by

$$reduction\;over\;\delta \;days\;=\;\frac{I_{control}^{ever}\left(T+D^{m}+\delta \right)\;-\;I_{mask}^{ever}\left(T+D^{m}+\delta \right)}{I_{control}^{ever}\left(T+D^{m}+\delta \right)\;-\;I_{control}^{ever}\left(T+D^{m}\right)}*100\%.$$ [1]

This equation produces the numbers we report to quantify the effects of face masks. *SI Appendix*, section D.2 describes our measures based on daily growth rates.

## Supplementary Material

Supplementary File

Supplementary File

## Data Availability

Public health data, including STATA and Matlab codes and data for replication purposes, have been deposited in FigShare (https://doi.org/10.6084/m9.figshare.13065920). All study data are included in the paper and *SI Appendix*.

## Footnotes

*The main channel through which masks reduce transmission of SARS-CoV-2 is the limiting effect for the spread of exhaled air, as argued by ref. 6. Refs. 6 and 7 argue that aerosols (as opposed to larger droplets) are filtered only by high-quality masks. Droplets are also filtered by home-made masks. Earlier work includes ref. 8 that was recently extended by ref. 9. Ref. 9 finds that all face covers (without an outlet valve) “reduce the front flow through jet by more than 90%.” As surgical and hand-made masks generally do not tightly fit, they generate backward and downward jets.

^†^Ref. 10 conducts a systematic review and meta-analysis. They do not report a study (see their table 1) that analyzes the entire population of a country.

^‡^As a measure for the quality of the fit between the treated region and its synthetic control group, the pretreatment root-mean-square prediction error (RMSPE) can be calculated and compared to a reference case. For Jena the pretreatment RMSPE is 3.145. This is considerably lower than an average RMSPE of 6.669 for all other 400 regions and their synthetic controls in the pretreatment period until 6 April. This points to the relatively good fit of the synthetic control group for Jena in this period.

^§^For a German-wide news report see, for example, ref. 14.

^#^See local newspaper reports, for instance ref. 15.

^ǁ^Further requirements, that are less central to our application, are listed in *Method and Data* and are discussed in *SI Appendix*, section B.

**We follow the method proposed in ref. 16 to calculate confidence intervals from P values. As pointed out in ref. 17, the interpretation of confidence intervals and P values is restricted to the question of whether or not the estimated effect of the actual treatment is large relative to the distribution of placebo effects.

^††^We analyze a measure that is introduced for the first time in this region. One might conjecture that our estimation measures both the true effect of a face mask but also any other change in behavior (washing hands, limiting interactions, staying at home more, etc.) that was triggered by this policy. This change in behavior is known as the Hawthorn effect. Individuals in this pioneer region might take the crisis more seriously than in the other areas. Although German health authorities had been strongly recommending such behavioral changes in daily life since mid-March, we cannot fully rule out this mixing of effects. Mobility data for federal states in *SI Appendix*, section C.6.2 show that federal states moved in a relatively coordinated way in this respect. Unfortunately, mobility data for Jena are not easily available.

^‡‡^Alternatively, we have also tested for pseudo-treatment effects in Jena over a period of 20 d before the introduction of face masks. This period is equally split into a pre- and pseudo posttreatment period. As *SI Appendix*, Fig. S11*B* shows, there is no strong deviation from the path of the synthetic control group.

^§§^This is perfectly in line with ref. 7 given the reduction in aerosols and droplets via using masks.

^##^We implicitly assume that compliance to rules in Germany is sufficiently homogenous. Some field observations in this respect would be very useful, especially across federal states in Germany and worldwide. Ref. 19 reports that compliance for distancing rules rises when masks are worn. As a first guess and assuming a compliance of 100% in our treated regions, one would expect that a reduction in compliance by x% of the population leads to a reduction of the effects of masks by x%.

***Common measures can be grouped inter alia into closures (of, e.g., restaurants or hotels, educational institutions or clubs, and leisure facilities), contact bans (for individuals, faith groups, or visits to hospitals or retirement homes), and contact rules (social distancing or quarantines after traveling).

^†††^We chose *T* = 29.5 as this yields a date when masks show an effect in the data on *T + D*^*m*^ = 40 where the epidemic is already beyond its peak in our simple model. This is consistent with Jena, where the incidence had already been declining when face masks became mandatory. Numerical solutions are computed in MATLAB. The code is available in *SI Appendix*.

## Change History

May 27, 2021: The SI Appendix and the Data Availability section have been updated.
