# Supplementary material for: Face masks considerably reduce COVID-19 cases in Germany
Source: Proc Natl Acad Sci U S A. 2020 Dec 3;117(51):32293–301. doi: 10.1073/pnas.2015954117 (PMC7768737; doi:10.1073/pnas.2015954117)
Supplement: Supplementary File [file pnas.2015954117.sapp.pdf]

PNAS
www.pnas.org

Supplementary Information for

Face Masks Considerably Reduce Covid-19 Cases in Germany

Timo Mitze[a], Reinhold Kosfeld[b], Johannes Rode[c] and Klaus Wälde[d]
(a) University of Southern Denmark, RWI and RCEA, (b) University of Kassel, (c) TU Darmstadt
(d) Johannes Gutenberg University Mainz, CESifo and Visiting Research Fellow IZA

November 13, 2020
(missing tick labels in figures S7, S13, S14 and S15 corrected on December 29, 2020)

Corresponding Author: Klaus Wälde

Email: waelde@uni-mainz.de

This PDF file includes:

- Supplementary text
- Figures S1 to S16
- Tables S1 to S14
- SI References

Other supplementary materials for this manuscript include the following:

a) STATA and Matlab codes
b) Data for replication purposes
c) Excel file delivering results in Table S13
All available at https://doi.org/10.6084/m9.figshare.13065920.v2

A Timing of public health measures and visibility in data

A.1 Timing of the introduction of mandatory face masks

Face mask were introduced in two ways in federal states. One measure relates to public transports and shops, the other to services for which a distance of 1.5 meters cannot be guaranteed. The points in time differ, however. An overview is in the next figure.

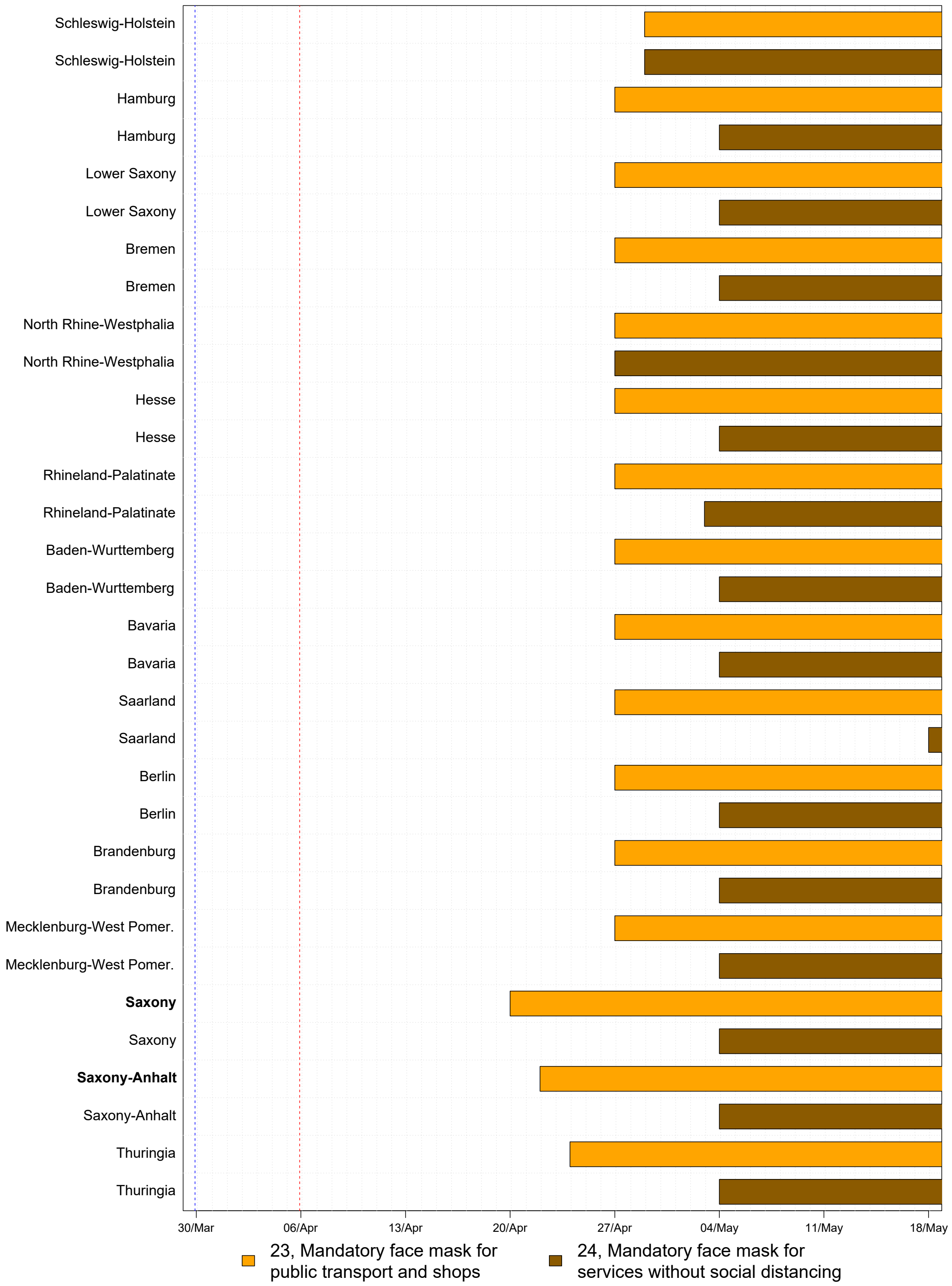

Figure S1: Time line of making face masks mandatory across federal states

We found two exceptions to this general principle of two measures. Thuringia only introduced face masks for public transports and shops. Bavaria introduced face masks for public transport

and shops first as a recommendation ("should be worn") on April 20. This was corrected by making face masks mandatory ("have to be worn") on April 27. We display the latter in the figure. We do not believe that adding Bavaria to the treatment group (by assuming that "should be" was already understood by the public as "have to be") would considerably change our findings.

For clarity, we present the dates for federal states in the following table. This table also displays regions such as Jena, which introduced face masks earlier than the federal state to which they belong.

Table S1: When face masks became compulsory in federal states and municipal districts

**federal state**	**public transport**	**services w/o distancing**	**individual NUTS3 region**	**mandatory face masks**	**difference in days to fed. state**
Baden-Wurttemberg	27.04.2020	04.05.2020	Landkreis Rottweil	17.04.2020	10
Bavaria	27.04.2020	04.05.2020			
Berlin	27.04.2020	04.05.2020			
Brandenburg	27.04.2020	04.05.2020			
Bremen	27.04.2020	04.05.2020			
Hamburg	27.04.2020	04.05.2020			
Hesse	27.04.2020	04.05.2020	Main-Kinzig-Kreis	20.04.2020	7
Mecklenburg-West Pomer.	27.04.2020	04.05.2020			
Lower Saxony	27.04.2020	04.05.2020	Wolfsburg	20.04.2020	7
			Braunschweig	25.04.2020	2
North Rhine-Westphalia	27.04.2020	27.04.2020			
Rhineland-Palatinate	27.04.2020	03.05.2020			
Saarland	27.04.2020	18.05.2020			
Saxony	20.04.2020	04.05.2020			
Saxony-Anhalt	22.04.2020	04.05.2020			
Schleswig-Holstein	29.04.2020	29.04.2020			
Thuringia	24.04.2020	-	Jena	06.04.2020	18
			Nordhausen	14.04.2020	10

A.2 The timing of other public health measures

As it is not enough to take only dates into account when face masks became mandatory, we provide an overview of the timing of other public health measures. This will show that our results capture the effects of face masks and not of other public health measures. Figure S2 shows the points in time when measures entered into force in Jena. All measures for Thuringia are also binding for Jena.[17] As Jena introduced three regulations concerning face masks, they became mandatory in three steps. April 1 saw the introduction of face masks for services where a distance of 1.5 meters cannot be kept. On April 6, masks became mandatory for public transports, shops, food deliveries stores and offices of craftsmen and service providers. As of April

[17] We are grateful to Jan Franke for many explanations related to public health measures in Jena and Thuringia.

10, masks also became mandatory at work and in public buildings, assuming a distance of 1.5 m could not be maintained. (See also the box on the next page.) Measures of April 1 and 6 are measures also employed by federal states subsequently (see SI Appendix, Section A.1). The measure of April 10 was employed only by Jena (at least in this wording).

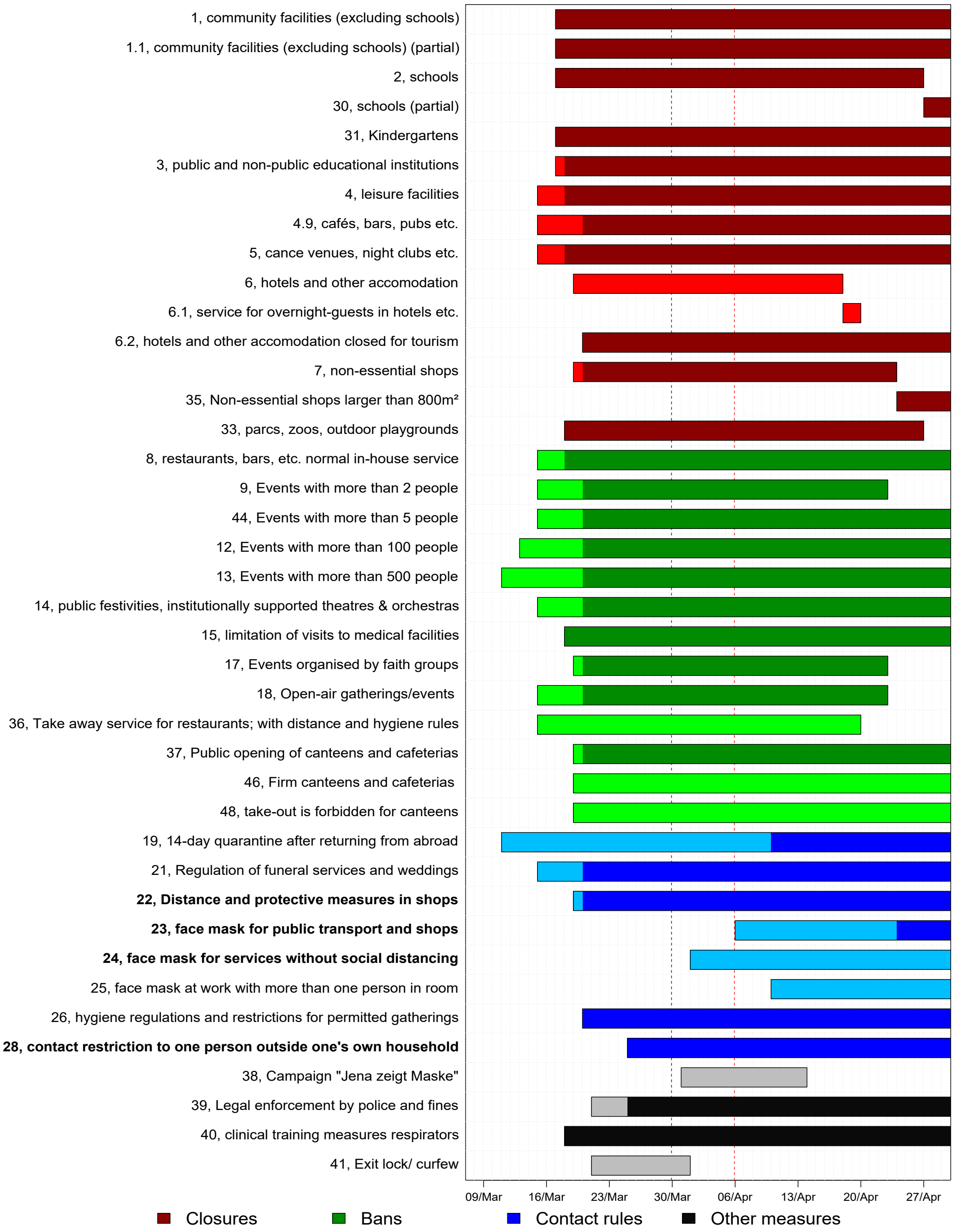

Figure S2: Time line of public health measures in Jena. Light bars indicate measures in force only in Jena, dark bars indicate measures in force in Thuringia (and thereby also in Jena)

Most importantly for our strategy to quantify the effect of face masks, we note that the regulation closest in time, apart from the campaign "Jena zeigt Maske", entered into force on March 25 (number 28, contact restriction). After face masks became mandatory, only exit strategies were implemented. Measure 6.1 that restricts service for over-night guests in hotels is part of

an exit strategies that allows hotels to reopen (measure 6) provided hotels do not provide service to over-night guests.[18]

This picture proves that there are no measures relevant for public health implemented in Jena that could affect the spread of Covid-19 around the time when face masks where introduced. We therefore conclude that it was indeed face masks whose effect we measured in the main text.

Due to the enormous interest in our study, both within Germany and worldwide, we reproduce here the regulation that makes face masks mandatory in Jena. The regulation is dated March 31, 2020 and enters in force on April 1, 2020.

Box S1: The regulation concerning face masks in Jena (source: Öffentliche Bekanntmachung der Stadt Jena, 31. 03. 2020, Vollzug des Gesetzes zur Verhütung und Bekämpfung von Infektionskrankheiten beim Menschen)

13. Jedermann hat bei Vorliegen der nachfolgend genannten Voraussetzungen einen Mund-Nasen-Schutz zu tragen. Anerkannt ist jeder Schutz, der aufgrund seiner Beschaffenheit geeignet ist, eine Ausbreitung von übertragungsfähigen Tröpfchenpartikeln durch Husten, Niesen, Aussprache zu verringern, unabhängig von einer Kennzeichnung oder zertifizierten Schutzkategorie (ausreichend sind daher auch aus Baumwolle selbstgeschneiderte Masken, Schals, Tücher, Buffs etc.)

a) Diese Verpflichtung gilt ab sofort für folgende Bereiche:
   - Die Inanspruchnahme und Erbringung von Dienstleistungen, bei denen sich der Mindestabstand von 1,5 m nicht durchgängig einhalten lässt.

b) Weiterhin gilt diese Verpflichtung ab dem 06.04.2020 für folgende Bereiche:
   - die Nutzung des öffentlichen Personennahverkehrs im Stadtgebiet Jenas,
   - das Betreten von geöffneten Verkaufsstellen,
   - das Betreten von Orten zur Abgabe von Speisen und Getränken zum Mitnehmen bzw. Ausliefern,
   - das Betreten der Diensträume von Handwerkern und Dienstleistern.

c) Schließlich gilt diese Verpflichtung ab dem 10.04.2020 für folgende Bereiche:
   - der Aufenthalt in geschlossenen Räumen mit mindestens einer anderen Person (insbesondere auch die Arbeitsstätte), ausgenommen hiervon ist der private Wohnbereich oder wenn im Raum pro Person mindestens 20 qm zur Verfügung stehen und der Mindestabstand von 1,5 m sichergestellt ist,
   - generell im öffentlichen Raum, wo eine Unterschreitung des Mindestabstands von 1,5 m nicht dauerhaft sichergestellt ist (dies gilt nicht bei Bewegung unter freiem Himmel, insbesondere Spazierengehen und Sport).

A.3 When are effects of public health measures visible in the data?

Imagine a public health measure is implemented on a certain day and that it is effective. When should we see the effects in the data? This delay between measure and statistical visibility depends on the usual incubation period and on the reporting delay. The incubation period is well-studied and has a median of 5.2 days and 95% of all delays lie in the range of around 2 to 12 days. They seem to be approximately log-normally distributed (1, 2). The reporting delay is not as well-studied. It consists of a delay due to diagnosis, testing and reporting of the test: A person with symptoms needs to decide to go to a general practitioner in order to obtain a diagnosis. With typical symptoms, a test is undertaken, and the result needs to be reported to the authorities. Formally, let D_I denote a random variable that describes the incubation period. Let D_R denote a second random variable that describes the delay between perceptible symptoms and reporting to authorities of a positive SARS-CoV2 test. We are interested in the distributional

[18] Note that measures 6 and 6.1 were implemented in Jena only (hence the light color). The corresponding measure 6.2 in Thuringia (dark red) closed hotels for tourism only.

properties of the overall delay defined as D = D_I+D_R. We will take the median of D as our measure for how long it takes before effects of public health measures are visible in the data.

Luckily, (3) provides information on the date of reporting and on the day of first symptoms (for around 80% of all reported Covid-19 cases). The difference between these two dates gives a vector of realizations of the random variable D_R. In total, we have 119,917 observations.

Findings for incubation. (1) and (2) describe the delay between infection and symptoms, i.e. the incubation period, by a lognormal distribution. To be precise about parameters in what follows, a lognormal distribution of a random variable X has the density $f(x) = \frac{1}{\sqrt{2\pi}\sigma x} e^{\left(-\frac{(lnx-\mu)^2}{2\sigma^2}\right)}$ $for\ x > 0$, where σ is the dispersion parameter and μ the scale parameter. The mean, median and variance are given by

$$E\ X = e^{\mu+\frac{\sigma^2}{2}}, m = e^{\mu},\ Var\ X\ =\ \left[e^{\sigma^2} - 1\right]e^{2\mu+\sigma^2}.$$

(2) report m=5.1 and that 95% of all cases lie between 2.2 and 11.5 days. The latter reads, more formally $\int_{2.2}^{11.5} f(x)dx = .95$. We numerically compute the parameters σ from this equation and obtain σ=0.4149. The scale parameter is given by μ=ln 5.1= 1.63.

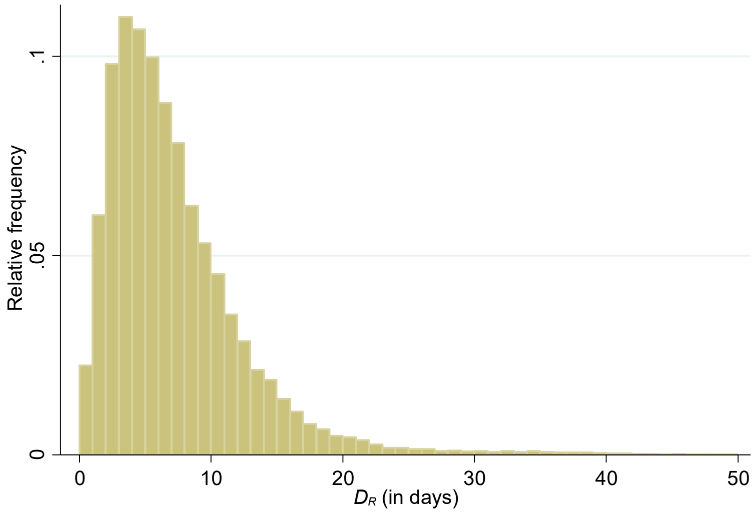

Notes: 0 ≤ D_R ≤ 50, Reporting day*(Meldedatum)* ≤ 6.5.2020

Figure S3: Histogram of delay between first symptoms and reporting

Findings for reporting. For illustration purposes, we plot a histogram of realizations of D_R in Figure S3. The mean, median (50% percentile), variance and standard deviation of D_R are reported in the next table.

Table S2: Descriptive statistics for the reporting delay D_R

**Mean**	**Median**	**Variance**	**Standard deviation**
6.80	6	30.92	5.56

Note: In the RKI data set (downloaded on June 7, 2020), there are 119,917 observations with information on day of infection (until reporting day May 6, 2020). We focus on 118,618 with D_R≥0.

Merging the two. We consider the duration between infection and reporting as one random variable. We call it total delay *D* and it consists of the sum of incubation and reporting delay, $D = D_I + D_R$. Obviously, the mean is $ED = ED_I + ED_R$ and the variance reads $VarD = VarD_I + VarD_R$ if we are willing to assume independence between the two random variables. As we do not believe that diagnosis or reporting lags are influenced by the length of the incubation period, we believe that this is a weak assumption.

As we need more information than the first two moments for our analysis, we now derive the distribution of *D*, i.e. the distribution of a sum of two random variables. We denote it by $F_D(\delta)$, i.e. $F_D(\delta)=Prob(D\leq\delta)$. We ask what the probability is that $D<\delta$ where δ is some constant. We continue to assume that D_I and D_R are independent random variables. The corresponding densities are $f(\delta_I)$ and $g(\delta_R)$, respectively. This probability is given by

$$Prob(D_I + D_R \leq \delta) = \int_0^{\delta}\left[\int_0^{\delta-\delta_I} f(\delta_I)g(\delta_R)d\delta_R\right]d\delta_I,$$

having the usual interpretation: when we are interested in values below or equal to δ, we let δ_I run from 0 to δ and δ_R from 0 to δ- δ_I such that the sum of the two is always smaller or equal to δ. Integrating over the joint density (which is a product given independence) gives the desired probability. This integral gives us the distribution $F_D(\delta)$ we were looking for.

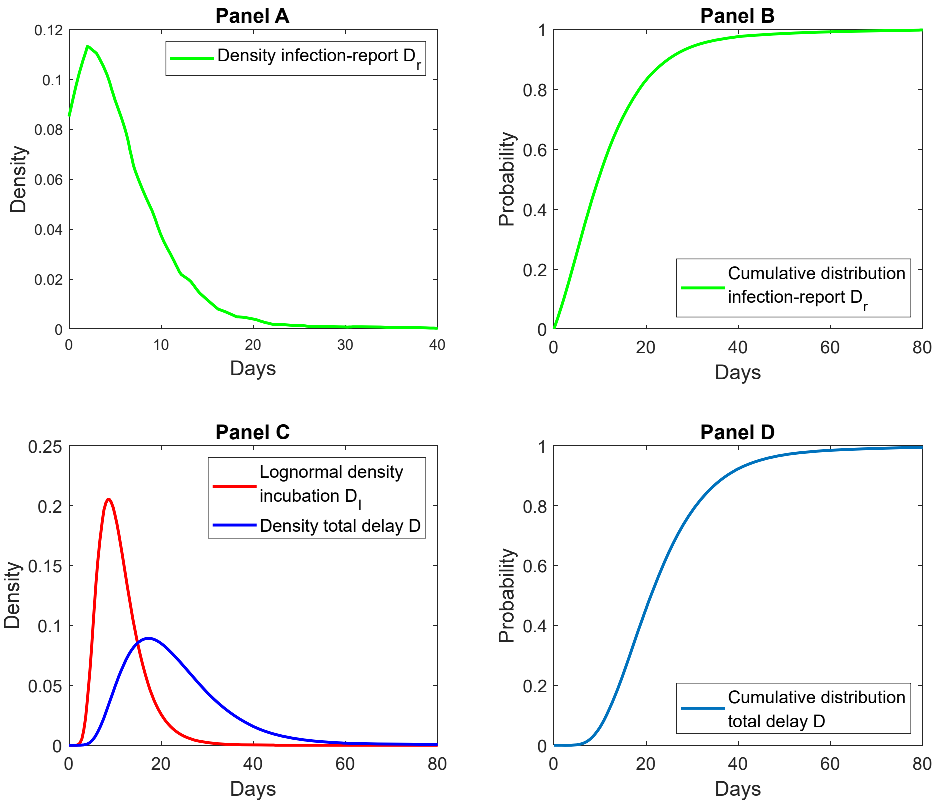

Figure S4: Density of the total delay D

If we needed a density $f_D(\delta)$, we could compute the derivative of this expression with respect to δ. This would give the usual convolution expression,

$$f_D(\delta) = \frac{dF_D(\delta)}{d\delta} = \frac{d}{d\delta}\int_0^{\delta}\left[\int_0^{\delta-\delta_I} f(\delta_I)g(\delta_R)d\delta_R\right]d\delta_I = \int_0^{\delta-\delta} f(\delta)g(\delta_R)d\delta_R + \int_0^{\delta}\frac{d}{d\delta}\left[\int_0^{\delta-\delta_I} f(\delta_I)g(\delta_R)d\delta_R\right]d\delta_I = \int_0^{\delta} f(\delta_I)g(\delta-\delta_I)d\delta_I.$$

Keeping in mind that we work with the assumption that $f(\delta_I)$ is the density of the exponential distribution and that $g(\delta_R)$ is the density corresponding to the histogram in Figure S3 above, we can easily compute the density numerically. Figure S4 provides a visual impression.

Our data imply a mean of 11.7 days and a median of 10.5 days. This provides a basis for studies (e.g., ref. 4) that need to assume a certain delay between infection and visibility in the data. [19] Our findings show that a delay of two to three weeks is too large. The percentiles of the total delay are in the following table.

Table S3: Percentiles of total delay *D*

**1**	**2.5**	**5**	**10**	**25**	**50**	**75**	**80**	**90**	**95**	**97,5**	**99**
3.42	4.09	4.78	5.70	7.65	10.52	14.30	15.41	18.74	22.22	26.29	34.23

A.4 Visibility in data II – Conceptual background

Conceptional background. We now present a standard SIR model. Let the (expected) number of individuals in the state of being susceptible at a point in time *t* be denoted by *S(t)*, the number of infectious individuals is *I(t)* and the number of removed is R(t).[20] The number of susceptible falls according to $\dot{S}(t) = -rI(t)S(t)$, where *r* is a constant and $rI(t)$ can be called the individual infection rate. Denoting the sum of individual recovery and death rate by a constant *a*, the number of infectious individuals changes according to $\dot{I}(t) = rI(t)S(t) - aI(t)$. Finally, the number of removed (recovered or death) individuals rises over time according to $\dot{R}(t) = aI(t)$. The number of individuals that have ever been infectious between the beginning of the epidemic in 0 and some point in time *t* amounts to $I^{ever}(\mathrm{t}) = \int_0^t rI(x)S(x)dx$. This number is the theoretical counterpart to the number of Covid-19 cases reported by health authorities worldwide. This model is used for our conceptional discussion in the main part of the paper.

We could also wish to inquire into the long-run effects of face masks. In this case, we would have to solve the underlying SIR model for the long-run, i.e. for when the epidemic is over. There are two issues. First, the future course of the epidemic is unknown given uncertainty about the availability of pharmaceutical solutions. Second, the long-run number of susceptible individuals depends on model parameters and can be larger than zero (8, 9). The SIR model therefore does not automatically end with herd immunity.[21] If the outflow from I(t) is larger than the inflow, the epidemic ends. To judge these long-run effects of face masks one would have to ignore potential pharmaceutical solutions and structurally estimate parameters of a much more elaborated SIR model. We therefore present the effects of masks by the measure proposed above in equation (1).

[19] We are grateful to Christof Kuhbandner for discussions of this point.
[20] More elaborate models designed for Covid-19 exist (e.g., ref. 5–7). The simple model employed here is, however, sufficient for our interpretation purposes.
[21] In this case, any public health intervention would only delay the epidemic but not reduce the long-run total number of infections. See e.g. (5, 6) for a discussion.

B Synthetic control method: Design, implementation and inference

This SI Appendix section provides further information on the design and implementation of the synthetic control method (SCM) to estimate the effect of face masks on the spread of Covid-19. We also explain how robustness tests can be used to conduct statistical inference.

Design. SCM is designed to run comparative case studies based on a data driven process. We have chosen SCM as the main vehicle for our empirical analysis here as the approach offers several attractive features relevant for our data settings. First, the scope of SCM to "*estimate the effects of <…> interventions that are implemented at an aggregate level affecting a small number of large units (such as cities, regions, or countries)*" (10, p. 3) clearly matches with our empirical setup. Compared to standard regression analyses used to identify treatment effects of a policy intervention (thereafter treatment), such as difference-in-difference (DiD) estimation, SCM is performs well when only one or very few units receive the treatment in focus (11, 12). Second, the method is flexible, transparent in terms of presenting the model fit and counterfactual of interest; it relies on relatively few requirements and has thus become a widely utilized tool in the policy evaluation literature (13) and for causal analyses in related disciplines (see, e.g., ref. 14, for an overview of SCM in health economics, 15, for a biomedical application).[22]

This section shall not serve the purpose of providing a formal description of the SCM approach (see ref. 10, 11, 17 for methodical details). The key *idea* of SCM is to establish a counterfactual that mimics a situation in which the treatment *ceteris paribus* would not have taken place. This is obtained by creating a synthetic control group out of the donor pool of control units (which have not received the treatment) and by comparing the outcomes of treated units and the synthetic control after the start of the treatment. Using a minimum distance approach, SCM selects weights for control units in the synthetic control group by relating treated and control units through a set of predictor variables measured prior to the start of the treatment. Weights for control regions are restricted to non-negative numbers and sum up to one in order to preclude extrapolation outside the support of the data.

To construct a suitable synthetic control group for our comparative case study of Jena, we need to find structurally similar regions in terms of their Covid-19 development before mandatory masks were introduced in Jena. Moreover, we need to make sure that face masks did not become mandatory in those regions during the treatment period. Such a control group would then most likely have had the same behavior as Jena in the absence of the mask obligation. We can then use this group to 'synthesize' Jena and conduct causal inference on the treatment effects of introducing face masks.

Synthetization means constructing a synthetic control group as weighted average of all regions in the donor pool of controls in which masks did not become compulsory earlier on. Regional weights are chosen in order to minimize a pre-treatment prediction error function for a set of predictor variables observed for Jena and the donor pool of control regions. Historical realizations of the outcome variable (cumulative number of Covid-19 cases; cumulative incidence rate) prior to the start of the treatment and several other regional characteristics (demography,

[22] SCM is employed by (16) to estimate the effect of the shelter-in-place order for California in the development of Covid-19. The authors find *inter alia* that around 1600 deaths from Covid-19 were avoided by this measure during the first four weeks.

health care system) comprise the set of predictors. The implementation of the SCM approach makes sure that the essential requirements are met.

Requirements and implementation. The effective use of SCM relies on certain contextual requirements (see ref. 10, 18, 19). These requirements, also listed in the Method and Data section in the main text, include that

(i) a donor pool of controls is available, i.e. not all regions receive the treatment during the period of the study,

(ii) data is available for a sufficiently long time period before and after the start of the treatment,

(iii) predictor values of the treated region are not extreme relative to those of controls, i.e. the treated region lies in the convex hull of control regions,

(iv) spillover effects of the treatment on controls are absent and

(v) there are no early anticipation effects, possibly related to concurrent policy interventions in the treated region.

In the empirical implementation of the SCM analysis, we ensure that these requirements are met as well as possible given the data at hand. The implementation is organized as follows. As baseline analysis, we focus on the single treatment case for the city of Jena. Our choice of Jena as essential comparative case study for the identification of treatment effects of mandatory face masks was made for several reasons, which can be directly linked to the aforementioned requirements. First, as shown in Figure 5 in the main text, Jena was the pioneer region for introducing face masks in public transport and sales shops on April 6. This results in a lead time of 18 days relative to mandatory face masks in the surrounding federal state Thuringia on April 24. By April 29, all German regions had introduced face masks. Referring to requirement i), a sufficiently long lag between the start of the treatment in Jena vis-à-vis control regions is important for effect identification as it ensures that a donor pool of controls without policy intervention is available.

Concerning the second requirement, our data sample for the daily development of (cumulative) Covid-19 cases throughout the period January 28 and May 11, 2020 allows us to operate with a sufficiently long pre- and treatment period. While we set the treatment period to the first 20 days after the introduction of face masks, the pre-treatment period covers the 14 days prior to the start of the treatment. The latter time window ensures that the highly dynamic nature of regional Covid-19 trajectories is properly captured for the construction of the synthetic control group. This helps to increase the fit between Jena and its synthetic control group in the pre-treatment period.

The synthetic control group is constructed by using the number of cumulative Covid-19 cases (measured one and seven days before the start of the treatment) and the number of newly registered Covid-19 cases (in the last seven days prior to the start of the treatment) as autoregressive predictor variables. As outlined above, the chosen lag structure will ensure that the highly dynamic Covid-19 development is properly captured. We use cross-validation tests to check the sensitivity of the SCM results when we impose a longer lag structure. The autoregressive predictors are complemented by cross-sectional data on the region’s demographic and

basic health care structure to control for confounding factors at the regional level. A list of variables together with summary statistics is given in SI Appendix, Section C.

With regard to the choice of predictor variables, SCM requires that values of predictor variables for the treated region are not extreme relative to those of controls, i.e. the treated region "lies in the convex hull" of control regions. As we show in greater detail in SI Appendix, Section C, Jena is in various ways a representative German city suitable for studying the Covid-19 development: On April 5, which is one day before face masks became compulsory in Jena, the cumulative number of registered Covid-19 cases in Jena was 144. This is very close to the median of 155 registered cases per region in Germany. Similarly, the cumulative number of Covid-19 incidences per 100,000 inhabitants was 126.9 in Jena compared to a mean value of 119.3 in Germany (compare Figure S6 in SI Appendix, Section C.2).

In order to preclude direct spillover effects (see requirement (iv)) from the treatment on non-treated control regions, we eliminate the immediate geographical neighbors of Jena from the donor pool. We also exclude those regions for which anticipation effects may have taken place because face masks became compulsory in quick succession to Jena (see Figure 5).

A further requirement for the effective use of SCM is that the timing of the introduction of face masks in Jena is not affected by concurrent public health intervention related to the Covid-19 spread. To support this requirement (v) from above, we have looked at all regulations (totaling almost 50) that were implemented in Jena between the beginning of March 2020 and end of April.[23] We have also looked at all regulations in Thuringia as these become automatically binding in Jena. A graphical illustration of the timing of the various measures and related discussion can be found in SI Appendix, Section A.1. As all other measures are more than 10 days away from masks becoming mandatory, we can be certain that we measure the effects of face masks.

In the implementation of our SCM analysis, we also run a series of sensitivity checks and placebo tests that will help to investigate the robustness of the results and check to what extent the underlying requirements of SCM are met. The results are reported in the main text. Important sensitivity checks are:

1. We account for early anticipation effects in Jena. Specifically, we take the announcement that face masks will become compulsory one week before their *de facto* introduction as an alternative start of the treatment period.[24]
2. We apply cross-validation tests to check for sensitivities related to changes in historical values in the outcome variables used as predictors. We also test for the sensitivity of the results when changing the composition of regions in the donor pool for computing the synthetic control group.
3. Significant public health measures that were introduced in Jena but not (or only slightly delayed) in the federal state of Thuringia or other German regions will be tested for their

---

[23] The first public health measure in Germany to mitigate the spread of Covid-19 dates from March 10 in North-Rhine Westphalia and prohibited meetings with more than 1000 participants. This measure was also implemented by many other federal states, including Thuringia one day after. See (20) for more background.

[24] We use March 30 as the day of the announcement when several local media reports covered the introduction of face masks on April 6. The general decree by the local administration in Jena was published on March 31.

intervening effect on the introduction of face masks by means of *placebo-in-time tests* and complementary *difference-in-difference regressions*.

We also run SCM analysis for a different outcome variable (cumulative incidence rate) and by disaggregating the data into specific age groups. A final important robustness test, which can also be used to conduct statistical inference in the SCM framework are *placebo-in-space tests*. We conduct such tests by estimating placebo treatment effects for each control region in which masks did not become compulsory early on. These placebo treatment effects treat each region in the donor pool of controls as treated region for the treatment period that applies for Jena. Using SCM, a synthetic control group is constructed for each control region (based on the same set of predictor variables as for Jena), and outcome differences between the region and its synthetic control measure the size of the placebo treatment effect. How these tests are employed to calculate p-values for assessing the significance of the estimated treatment effects is described below.

Inference. The implementation of comprehensive *placebo-in-space tests* allows us to conduct statistical inference on the basis of permutation tests as suggested by (18) and applied, for example, by (21) or (22). Statistical significance is established by comparing the difference of outcomes between the treated region and its synthetic control group and the differences among donor regions and their respective synthetic control groups. For a significant treatment effect, we expect that the estimated placebo treatments for control regions are small (or even reversed) relative to those for the treated region. We calculate time-specific significance levels for the test of the hypothesis that mandatory face masks did not significantly reduce the number of reported Covid-19 cases for each day after the treatment.

The (pseudo) *p*-values reported in the main text are derived from a ranking of the actual treatment effect within the distribution of placebo treatment. We follow the suggestion in (23) and compute adjusted *p*-values taking the pre-treatment match quality of the placebo treatments into account. The refined procedure draws inference only from donors with a good fit in the pre-treatment period. Specifically, we do not include placebo effects in the pool for inference if the match quality of the control region, measured in terms of the pre-treatment root mean squared prediction error (RMSPE), is greater than 20 times the match quality of the treated unit. Further, we adjust *p*-values by dividing all underlying effects with the corresponding pre-treatment match quality.[25] We finally use the set of *p*-values to compute confidence intervals for treatment effects to visualize the significance and precision of the estimated effects (25).

Multiple treatment analysis. Although the case study of Jena can be framed in a clear identification strategy, the Covid-19 spread in a single municipality may still be driven by certain particularities and random events. This may prevent a straightforward generalization of estimated effects. We therefore also test for treatment effects in regions that introduced face masks after Jena but still before they became compulsory all across Germany. To do so, we extend the single treatment approach to the analysis of multiple treated units by considering all regions in the treated group that introduced face masks by April 22. This results in a total of 32 regions, of which 8 are larger cities (*kreisfreie Städte*).

All other regions apart from those located in Thuringia (April 24) and Schleswig-Holstein (April 29) introduced face masks on April 27. We employ this staggered introduction to study the

[25] We conduct all estimations in STATA using "Synth" and "Synth Runner" packages (23, 24). Data and estimation files are available on the journal's web page.

effects of mandatory masks up to May 11, which gives us a time window of 20 days to measure treatment effects. We end our analysis on May 11 to avoid a potential underestimation of treatment effects since by that day all control regions had had face masks in use for 14 days. This cut-off date is important as we expect that differences in the epidemic spread between treated and control regions would disappear afterwards if we assume a median incubation period of 5.2 days (see ref. 1, 2) and a similar reporting lag. This overall time lag between the infection with SARS-CoV-2 and registration in the data is also crucial for the interpretation of our results and we discuss it in detail in SI Appendix, Section A.3.

SCM and difference-in-difference estimation. We use SCM and DiD regressions as complementary estimation approaches to identify the treatment effects of face masks. The choice of SCM as our main vehicle of analysis results from the different requirements of both estimators. Regression approaches such as DiD usually perform poorly when the treatment group is very small. Moreover, the identification of treatment effects in the DiD approach strongly relies on the validity of the parallel trend assumption. A parallel trend in the outcome variable between the treated and control units ascertained in the pre-treatment period is assumed to hold in the treatment period in the counterfactual case without the intervention. For establishing the treatment effect by SCM, the parallel trend assumption need not to imposed (11, 14, 17). This is due to the construction of the SCM, in which common trends between the treated and similar control regions are favorable for finding an appropriate counterfactual trajectory. While DiD accordingly associates equal weights to all control regions, SCM replaces equal weights by optimal weights. Optimal weights minimize the distance between treated and control regions in terms of pre-intervention characteristics including lagged outcome values.

However, DiD estimation also has certain advantages over SCM. These particularly relate to the estimation of dynamic treatment effects. We accordingly use DiD regressions in order to check the robustness of our empirical results against the potential presence of effects stemming from latent policy interventions or other events that may affect the outcome variable (see SI Appendix, Section F for a detailed description of these robustness tests).

C Data description and additional SCM estimation results for Jena

C.1 Summary statistics for outcome and predictor variables

The Robert Koch Institute (RKI) collects data on registered Covid-19 cases from local health authorities in the individual municipal districts (NUTS3 regions) in Germany and provides updates to this database on a daily basis (available via API). We use the cumulative number of registered Covid-19 cases in each municipal district as main outcome variable.[26] As an alternative outcome variable, we also employ the cumulative incidence rate, i.e. the number of cumulative Covid-19 cases per 100,000 inhabitants. Summary statistics for these two outcome variables together with information on the daily number of newly registered Covid-19 cases are shown in Panel A of Table S4.

[26] We are aware of the existence of hidden infections. As it appears plausible to assume that they are proportional to observed infections across regions, we do not believe that they affect our results. We chose the date of reporting (as opposed to date of infections) because not all reported infections include information about the date of infection.

Table S4: Summary Statistics of Covid-19 indicators (outcome variables) and predictors characterizing the regional demographic structure and basic health care system

	**Mean**	**S.D.**	**Min.**	**Max.**
**PANEL A: Data on registered Covid-19 cases**				
[1] Newly registered cases per day	3.91	10.24	0	310
[2] Cumulative number of cases	147.70	327.12	0	6066
[3] Cum. cases [2] per 100,000 inhabitants	73.50	120.38	0	1542.69
**PANEL B: Regional demographic structure and local health care system**				
Population density (inhabitants/km^2)	534.79	702.40	36.13	4,686.17
Population share of highly educated* individuals (in %)	13.07	6.20	5.59	42.93
Share of females in population (in %)	50.59	0.64	48.39	52.74
Average age of females in population (in years)	45.86	2.11	40.70	52.12
Average age of males in population (in years)	43.17	1.83	38.80	48.20
Old-age dependency ratio (persons aged 65 years and above per 100 of population aged 15-64 years)	34.34	5.46	22.40	53.98
Young-age dependency ratio (persons aged 14 years and below per 100 of population aged 15-64 years)	20.54	1.44	15.08	24.68
Physicians per 10,000 of population	14.58	4.41	7.33	30.48
Pharmacies per 100,000 of population	27.01	4.90	18.15	51.68
Settlement type (categorial variable$)	2.59	1.04	1	4

Notes: * = International Standard Classification of Education (ISCED) Level 6 and above; $ = included categories are 1) larger cities *(kreisfreie Großstädte)*, 2) urban districts *(städtische Kreise)*, 3) rural districts *(ländliche Kreise mit Verdichtungsansätzen)*, 4) sparsely populated rural districts *(dünn besiedelte ländliche Kreise)*.

The variables presented in Table S4 are also used as predictor variables in the SCM approach. Selection of the latter has been guided by their ability to describe the regional number and dynamics of reported Covid-19 cases in the treatment period. Obviously, past values of (newly) registered Covid-19 cases are important for predicting regional trajectories of Covid-19 cases over time in an autoregressive manner. In addition, we argue that a region's demographic structure, such as the overall population density and age structure, and its basic health care system, such as the regional endowment with physicians and pharmacies per population, are important factors for characterizing the local context of Covid-19. These latter regional predictor variables are only available as annual averages and are obtained from the INKAR online database of the Federal Institute for Research on Building, Urban Affairs and Spatial Development (26). We use the latest year available in the database, which is 2017. Despite this time lag, we argue that regional demographic structures only gradually vary over time such that they can be used to proxy regional differences during the spread of Covid-19 in early 2020.

C.2 Trajectories of cumulative Covid-19 cases and box plots for predictor variables

As outlined in the main text and in SI Appendix, Section B, one requirement of SCM is that predictor values of the treated region are not extreme relative to those of controls. This SI Appendix section goes beyond descriptive statistics by visualizing the overlap in variable values for Jena and the donor pool. We do so for both outcome and predictor variables. First, Figure S5 plots the cumulative number of Covid-19 cases for Jena and the (daily) 2nd to 4th quintile of

the control regions. As Jena lies well within the control regions, its infection dynamics was not extreme relative to the donor pool in the pre-treatment period.

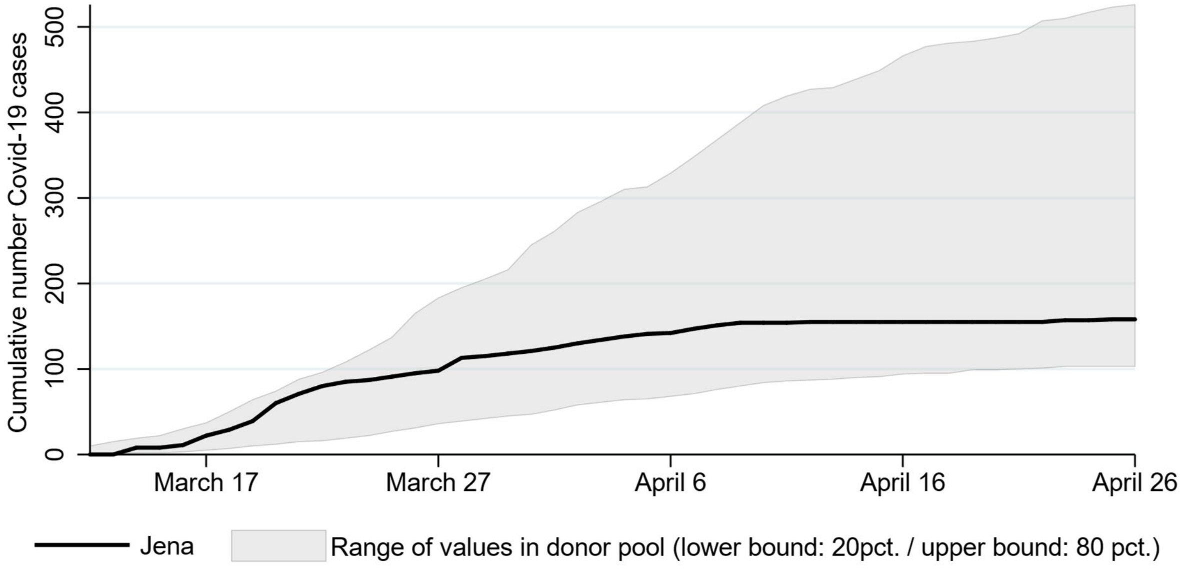

Figure S5: Overlap of cumulative Covid-19 cases between Jena and the donor pool of control regions

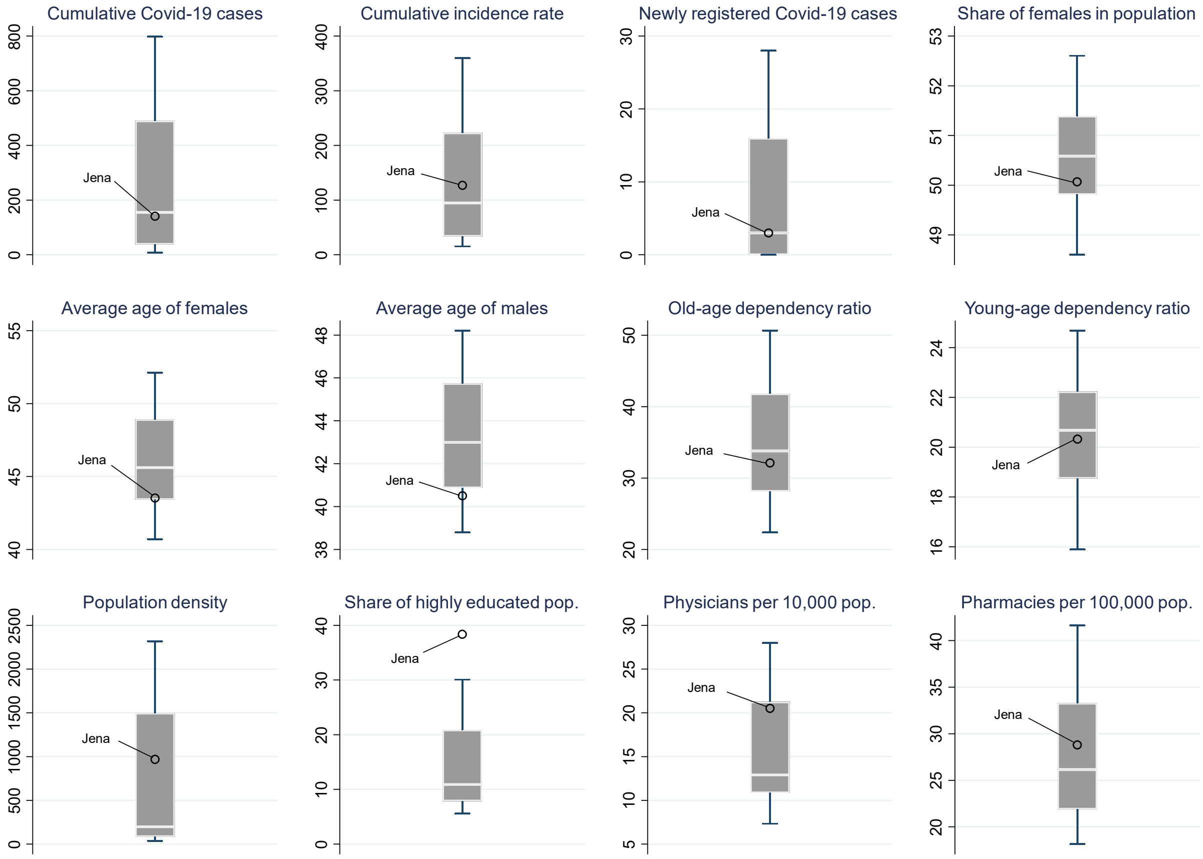

Figure S6: Box plots for the regional distribution of predictor variables (donor pool of controls and Jena).

Notes: Box plots are drawn to cover 90% of values for regional distribution across German regions; further outliers are ignored. For time-varying predictors, the regional distribution on April 5 is shown in the box plots.

Box plots in Figure S6 lend additional support for perceiving Jena as an "average region" in Germany. Infection level and dynamics prior to the treatment period on April 5 were very close to the median value of the donor pool (as shown for the cumulative number, the cumulative incidence rate and the number of new Covid-19 cases). Other predictor values (as summarized in Panel B of Table S4) also display a sufficient overlap between Jena and the control regions. Only the share of highly educated individuals is exceptional, probably reflecting the status of Jena as a university town with roughly 18,000 students out of approximately 108,000 inhabitants.

To test whether this exception drives our results, we have performed cross-validation tests that sequentially exclude individual variables from the set of predictors. As shown in SI Appendix, Section C.7, the results for the different SCM specifications do not show structural differences and identify similar treatment effects. Our results are therefore not driven by this one exception.

C.3 Control regions, SCM weights and pre-treatment predictor balance

This SI Appendix section provides details on the composition of the synthetic control group used to identify treatment effects of face masks in Figure 1 in the main text. Balancing properties of the SCM approach together with the root mean square percentage error (RMSPE) as a measure for the quality of the pre-treatment fit between Jena and its synthetic control group are also reported.

The donor pool used to construct the synthetic control group includes all other German NUTS3 regions except for the two immediate neighboring regions of Jena (Weimarer Land, Saale-Holzland-Kreis) and the regions Nordhausen and Rottweil. The latter two introduced face masks in rapid succession to Jena on April 14 and April 17, respectively.

Table S5: Composition of synthetic control group for Jena with associated SCM weights

**Introduction of face masks (Panel A in Figure 1)**		
**ID**	**NUTS 3 region**	**Weight**
13003	Rostock	0.326
6411	Darmstadt	0.311
3453	Cloppenburg	0.118
7211	Trier	0.117
6611	Kassel	0.082
5370	Heinsberg	0.046

Notes: Donor pools corresponds to SCM estimation in Panel A of Figure 1. Sample weights are chosen to minimize the RMSPE ten days prior to the start of the treatment.

Table S6: Pre-treatment predictor balance and RMSPE for SCM in Figure 1

**Treatment:**		**Introduction**		**Announcement**
	Jena	Synthetic	Jena	Synthetic
Cumulative number of registered Covid-19 cases (one and seven days before start of treatment, average)	129.5	129.2	93	92.7
Number of newly registered Covid-19 cases (last seven days before start of the treatment, average)	3.7	3.8	5	5.2
Population density (Population/km^2)	968.1	1074.3	968.1	947.9
Share of highly educated population (in %)	38.4	22.8	38.4	26.3
Share of females in population (in %)	50.1	50.1	50.1	50.1
Average age of female population (in years)	43.5	43.7	43.5	43.9
Average age of male population (in years)	40.5	40.6	40.5	40.8
Old-age dependency ratio (in %)	32.1	29.3	32.1	29.8
Young-age dependency ratio (in %)	20.3	19.6	20.3	19.5
Physicians per 10,000 of population	20.5	19.8	20.5	20.8
Pharmacies per 100,000 of population	28.8	28.7	28.8	28.6
Settlement type (categorial variable)	1	1.3	1	1.9
**RMSPE (pre-treatment)**	**3.145**		**4.796**	

C.4 SCM results by age groups

We refined our Jena analysis also for different age groups. We looked at inhabitants aged 15 to 34, 35 to 60 and above 60. We again studied the treatment effect for the implementation date for masks on April 6 and for the announcement of masks on March 30.

Predictor variables are chosen as for the baseline specification shown in Figure 1 in the main text. Results visible in the next figure are also discussed in the main text. Regions in the synthetic control groups corresponding to the three age groups are listed in Table S7.

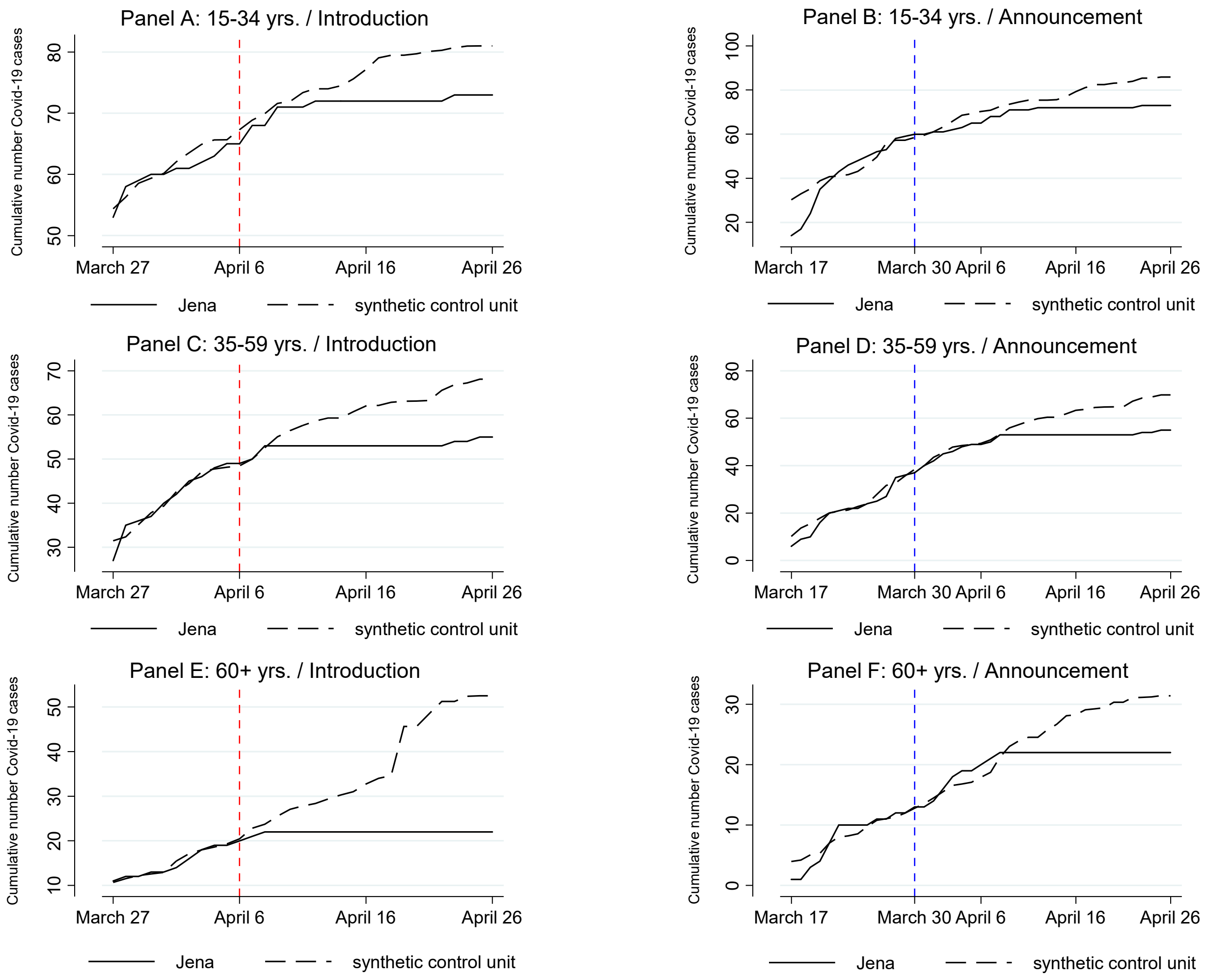

Figure S7: Treatment effects for introduction and announcement of face masks in Jena by age groups

Table S7: SCM weights for control regions used to construct synthetic Jena for three age groups

Age Group 15-34 years			Age Group 35-59 years			Age Group 60 years and above		
**ID**	**NUTS 3 region**	**Weight**	**ID**	**NUTS 3 region**	**Weight**	**ID**	**NUTS 3 region**	**Weight**
1001	Flensburg	0.323	6411	Darmstadt	0.528	6411	Darmstadt	0.522
7211	Trier	0.207	16055	Weimar	0.16	16055	Weimar	0.244
13003	Rostock	0.184	14511	Chemnitz	0.15	7316	Neustadt a.d. Weinstraße	0.068
5370	Heinsberg	0.142	8221	Baden-Baden	0.07	9562	Erlangen	0.06
3453	Cloppenburg	0.107	6434	Hochtaunus-kreis	0.062	3356	Osterholz	0.056
6413	Offenbach am Main	0.038	8435	Bodenseekreis	0.029	5515	Münster	0.027
			5370	Heinsberg	0.001	9188	Starnberg	0.022

Notes: Sample weights are chosen to minimize the RMSPE ten days prior to the start of the treatment.

C.5 Effects on cumulative number of infections per 100,000 inhabitants

One might be concerned that absolute infection numbers are not appropriate as regions differ in size measured by number of inhabitants. We checked our results by re-estimating effects for incidence, i.e. infections normalized by the size of the population. Our outcome variable is then given by cumulative reported infections divided by population size and multiplied by 100,000. Predictor variables are chosen as for the baseline specification shown in Figure 1.

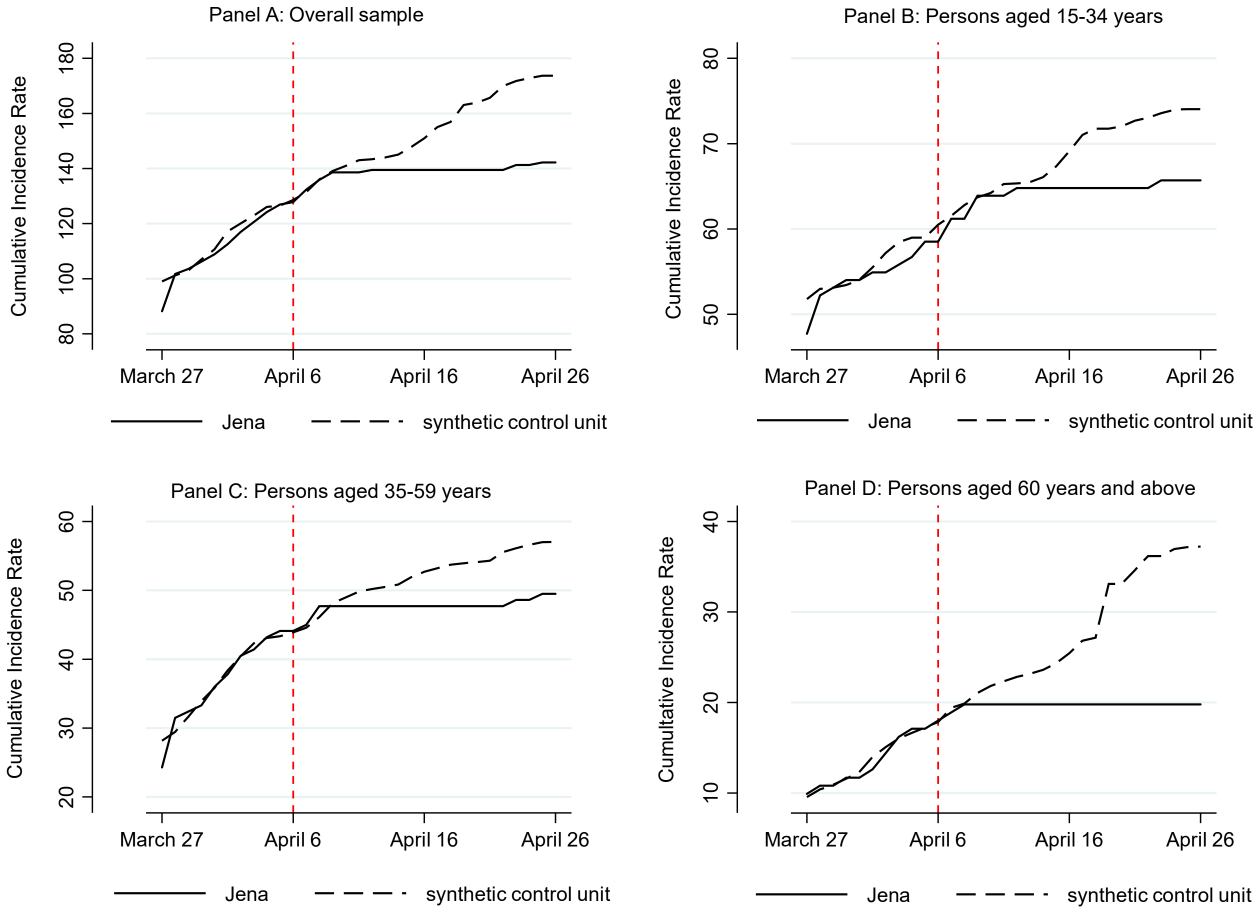

Figure S8: Treatment effects for introduction of face masks on cumulative incidence rate

Table S8: SCM weights for control regions used to construct synthetic Jena (cumulative incidence rate)

ID	NUTS 3 region	Weight
6411	Darmstadt	0.46
15003	Magdeburg	0.171
5370	Heinsberg	0.133
13003	Rostock	0.093
5515	Münster	0.066
11000	Berlin	0.035
12052	Cottbus	0.032
6611	Kassel	0.011

Note: Synthetic control group corresponds to SCM estimation in Figure S8. Sample weights are chosen to minimize the RMSPE ten days prior to the start of the treatment.

Table S9: SCM weights for control regions used to construct synthetic Jena (cumulative incidence rate; by age groups)

Age Group 15-34 years			Age Group 35-59 years			Age Group 60 years and above		
**ID**	**NUTS 3 region**	**Weight**	**ID**	**NUTS 3 region**	**Weight**	**ID**	**NUTS 3 region**	**Weight**
5370	Heinsberg	0.377	6411	Darmstadt	0.419	6411	Darmstadt	0.448
13003	Rostock	0.288	14511	Chemnitz	0.184	14612	Dresden	0.313
1001	Flensburg	0.14	14612	Dresden	0.154	9188	Starnberg	0.071
6611	Kassel	0.138	8221	Heidelberg	0.138	16054	Suhl	0.069
11000	Berlin	0.058	9188	Starnberg	0.088	5515	Münster	0.06
			5370	Heinsberg	0.016	8221	Heidelberg	0.039

Notes: Donor pools corresponds to SCM estimations in Figure S8. Sample weights are chosen to minimize the RMSPE ten days prior to the start of the treatment.

C.6 Announcement and mobility

C.6.1 Google trends and announcement effects

To understand the role of a potential announcement effect or the effect of the information campaign in Jena for masks, we looked at search intensities in the internet. Time series are displayed in the next figure, results are discussed in the main text.

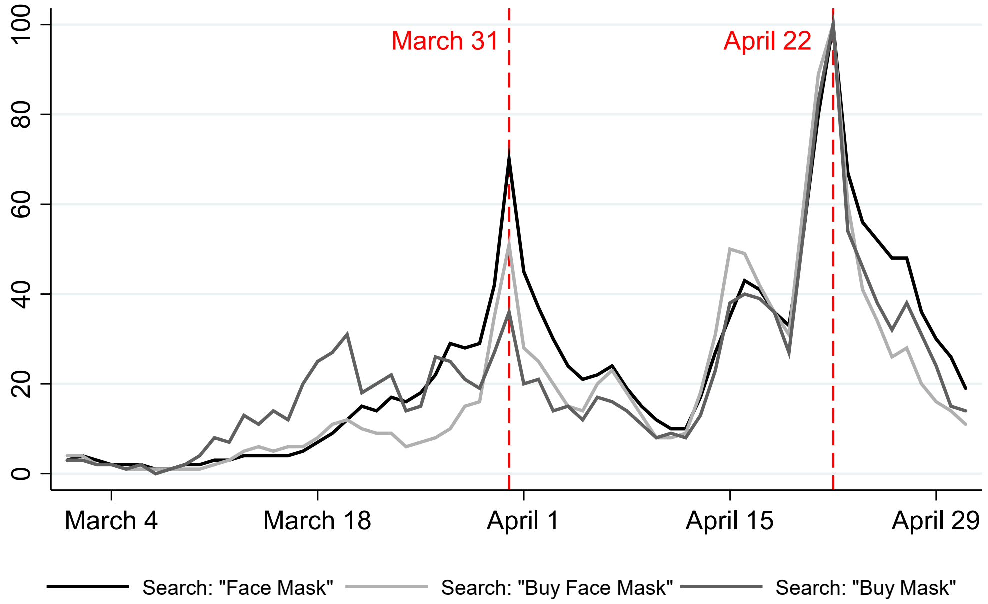

Figure S9: Online search for face masks and purchase options according to Google Trends

Notes: Online search for keywords (in German) as shown in the legend as Face Mask ("Mund-Nasen-Schutz"), Buy Face Mask ("Mundschutz kaufen") and Buy mask ("Maske kaufen"); alternative keywords show similar peaks but with a lower number of hits; based on data from Google Trends (27).

C.6.2 Mobility trends across German federal states

Figure S10 shows overall mobility patterns across German federal states between Feb 17 and May 18, 2020 based on Google (28). The data track the frequency of visits to different places covered in Google maps on a daily basis compared to a baseline. The latter is set as median value for the corresponding weekday during Jan 3 and Feb 6, 2020. To arrive at a compact

measure of regional mobility, we aggregated data over the different place categories: retail and recreation, groceries and pharmacies, parks, transit stations and workplaces. Given the high volatility of daily data, Figure S10 displays weekly averages. The mobility trends show a clear common pattern: With public health measures taken across all federal states to restrict professional and social contacts (RSC), mobility sharply declined in mid-March. It stayed low for most of the following weeks and only gradually increased from mid-April onwards when first actions to lift RSC and to re-open the economy have been taken (see ref. 20 for details).

Importantly, during the timing of the mandatory introduction of face masks in Jena on April 6, no significant change in mobility patterns across federal states can be observed, which potentially confounds our empirical estimates. Although mobility data are increasingly used to study the effects of public health measures, the inspection of the Google data urges us to use such data only very carefully in comparative studies at the countries/regional level given a the generally high volatility and significant outliers. This is also recognized by (28).[27]

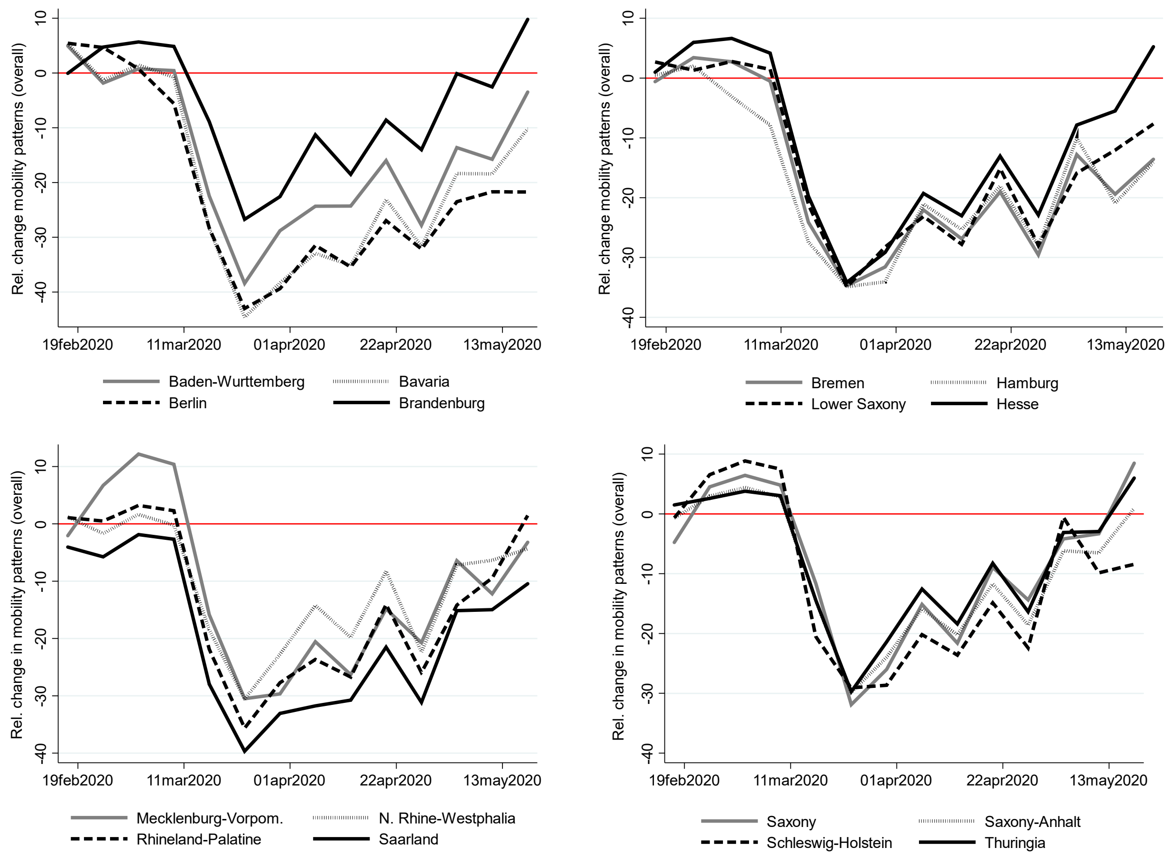

Figure S10: Trend in mobility patterns across German federal states (Feb 17 to May 18, 2020) from (28).

[27] For details see (29).

C.7 Cross validation and additional placebo-in-time test

The cross-validation and additional placebo-in-time tests reported in Figure S11 check the robustness of the estimated treatment effects for alterations to our benchmark SCM specification. The cross-validation tests in Panel A modify the lag structure of the included time-varying predictor variables. The additional placebo-in-time test in Panel B checks for anticipation effects 20 days before the start of the actual treatment on April 6 (equally split into a pre- and treatment period from March 26 onwards).

In Panel A the baseline specification for the synthetic control uses both the number of cumulative Covid-19 cases (measured one and seven days before the start of the treatment) and the number of newly registered Covid-19 cases (in the last seven days prior to the start of the treatment) as predictor variables. The alternative specifications lag both of these variables simultaneously by 1, 3 and 7 days.

Panel B displays pseudo-treatment effects for Jena over a period of 20 days before the introduction of face masks. This period is equally split into a pre- and pseudo post-treatment period.

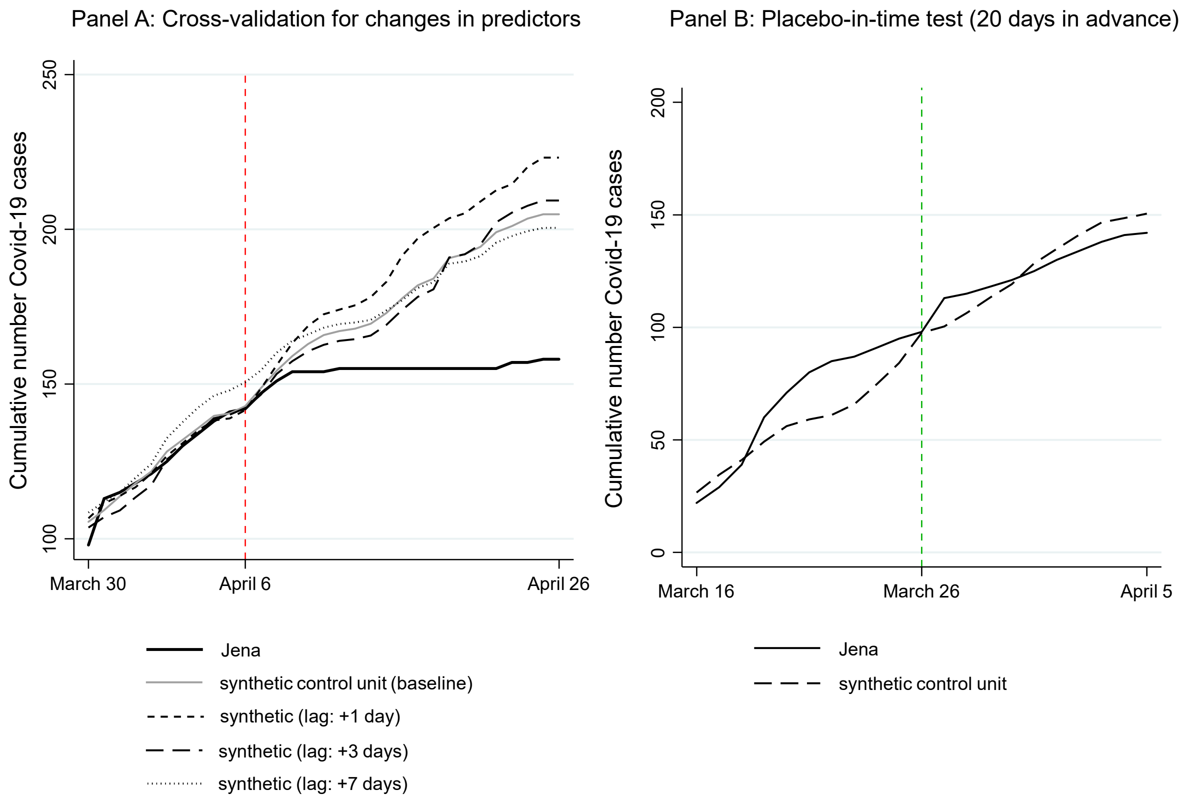

Figure S11: Cross-validation test for changes in time-varying predictor variables and placebo-in-time test

Figure S12 shows the results for an additional cross-validation test, which sequentially excludes time-constant predictors from the full set of predictor variables. The estimated trajectories for the respective synthetic control groups excluding a certain predictor follow a very similar trend and all identify a reduction in the number of cumulative Covid-19 cases in Jena vis-à-vis the synthetic Jena that widens over time. Excluding population density implies larger outcome difference than for other excluded variables. Most importantly, however, excluding the share of skilled individuals, the variable by which Jena seems exceptional according to Figure S6, does

not affect our main finding in any essential way. We therefore argue that using the full set of predictors is the most reasonable approach to identify reliable treatment effects.

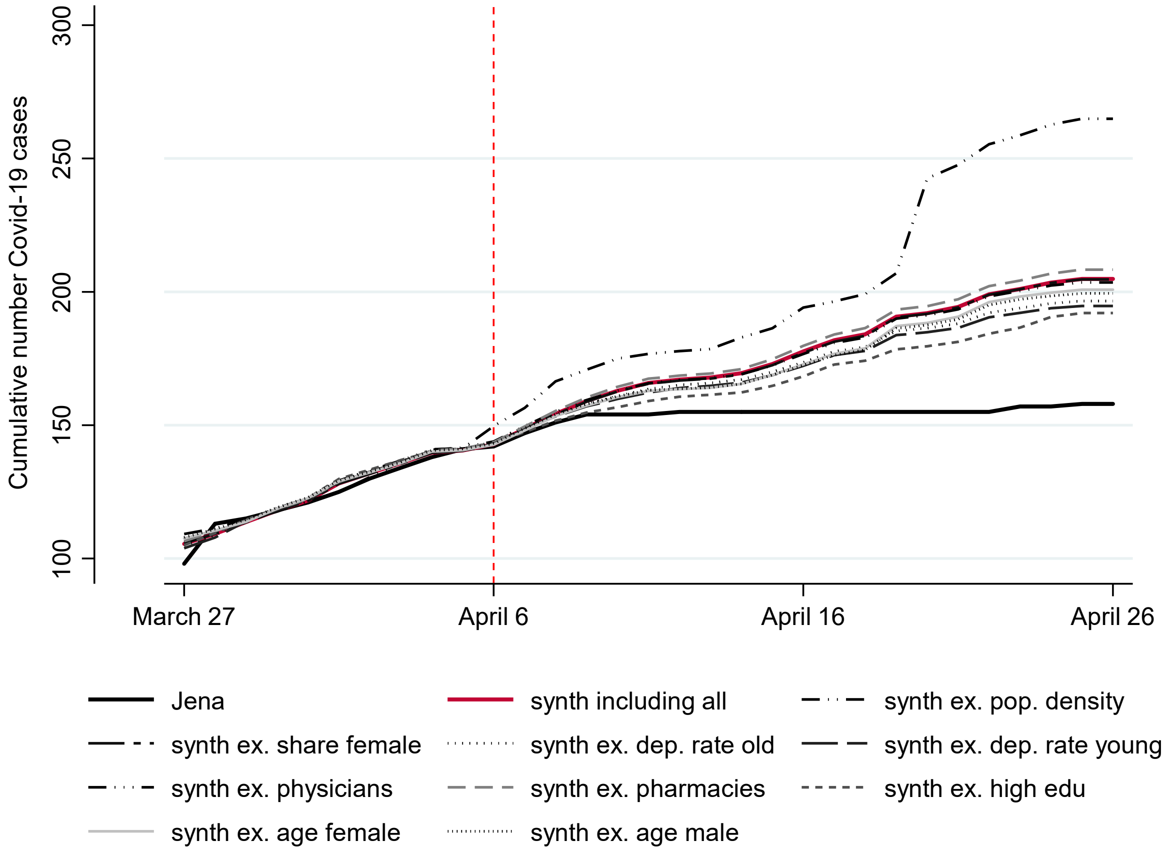

Figure S12: Cross-validation test for changes in the set of time-constant predictors

C.8 Changes in donor pool for synthetic Jena

A further robustness check changes the donor pool from which synthetic Jena is constructed (see main text for detailed definition). Infection dynamics in the various synthetic groups are shown in the next figure. Again, predictor variables are chosen as for baseline specification shown in Figure 1.

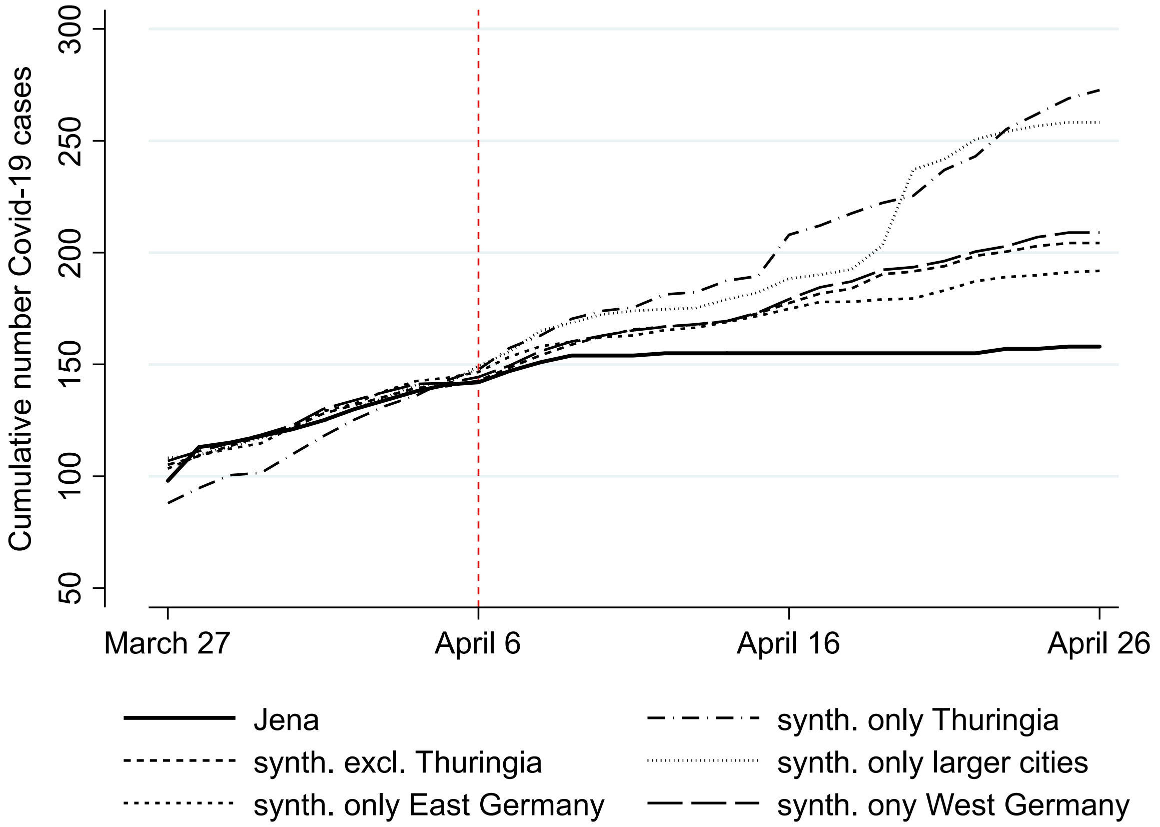

Figure S13: Treatment effects for changes in donor pool used to construct synthetic Jena

Table S10: Weights for control regions used to construct synthetic Jena for various donor pools

	**Only Thuringia**			**Excluding Thuringia**			**Only larger cities**	
**ID**	**NUTS 3 region**	**Weight**	**ID**	**NUTS 3 region**	**Weight**	**ID**	**NUTS 3 region**	**Weight**
16076	Greiz	0.533	13003	Rostock	0.318	6411	Darmstadt	0.504
16051	Erfurt	0.467	6411	Darmstadt	0.302	13003	Rostock	0.304
			7211	Trier	0.129	5113	Essen	0.192
			3453	Cloppenburg	0.122			
			6611	Kassel	0.083			
			5370	Heinsberg	0.046			
	**Only East Germany**			**Only West Germany**				
**ID**	**NUTS 3 region**	**Weight**	**ID**	**NUTS 3 region**	**Weight**			
16051	Erfurt	0.865	6411	Darmstadt	0.242			
14612	Dresden	0.124	3402	Emden	0.198			
11000	Berlin	0.011	6611	Kassel	0.169			
			7211	Trier	0.168			
			4012	Bremerhaven	0.167			
			5370	Heinsberg	0.057			

Note: Donor pools corresponds to SCM estimations in Figure S13. Sample weights are chosen to minimize the RMSPE ten days prior to the start of the treatment.

C.9 Place-in-space tests for other major cities in Thuringia

The placebo-in-space tests for other cities and larger regions in Thuringia uses the same set of predictors as for Jena (see Figure 1). The reported regions cover all *kreisfreie Städte* plus Gotha (*Landkreis*). The cities Weimar, Suhl and Eisenach have been aggregated since the absolute number of reported Covid-19 is low in these cities, which made it hard to find a suitable control group. None of these regions shows a reduction in the number of Covid-19 cases as in Jena. This confirms that latent macro-effects, for example infection dynamics or other interdependencies in Thuringia as a whole, are not behind the measured treatment effects for Jena.

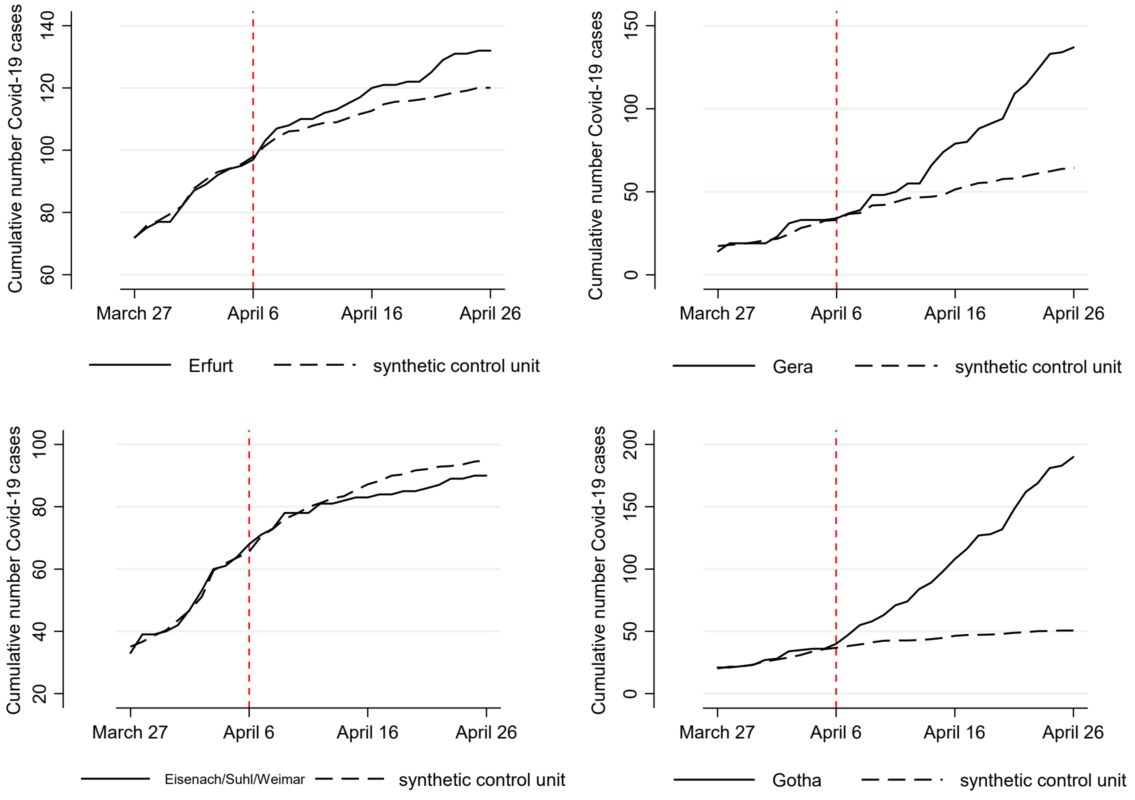

Figure S14: Placebo tests for the effect of face masks in other cities in Thuringia on April 6.

As a side note, the cumulative number of Covid-19 cases in Gotha relative to its synthetic control group stands out here. Local newspapers report[28] that there were outbreaks in two hospitals and three retirement homes. The fast rise in Gotha can therefore best be explained by a bad realization of an otherwise normal stochastic process. One could relate the outbreaks to issues with compliance but – to the best of our knowledge – data are not available in this respect.

[28] See e.g. (30).

Table S11: SCM weights for control regions in synthetic control groups (other cities in Thuringia)

	Erfurt			Gera	
**ID**	**NUTS 3 region**	**Weight**	**ID**	**NUTS 3 region**	**Weight**
13003	Rostock	0.28	15001	Dessau-Roßlau	0.501
16055	Weimar	0.244	16054	Suhl	0.222
3356	Osterholz	0.212	7318	Speyer	0.162
7313	Landau in der Pfalz	0.154	8231	Pforzheim	0.061
6413	Offenbach am Main	0.078	7311	Frankenthal (Pfalz)	0.046
5370	Heinsberg	0.029	8211	Baden-Baden	0.005
5515	Münster	0.004	9662	Schweinfurt	0.003
			14521	Erzgebirgskreis	0.001
	**Weimar/Suhl/Eisenach**			**Gotha**	
**ID**	**NUTS 3 region**	**Weight**	**ID**	**NUTS 3 region**	**Weight**
15001	Dessau-Roßlau	0.263	15081	Altmarkkreis	0.23
12052	Cottbus	0.236	16077	Altenburger Land	0.164
13004	Schwerin	0.202	15086	Jerichower	0.161
9361	Amberg	0.177	3402	Emden	0.111
14626	Görlitz	0.069	16071	Weimarer Land	0.108
9363	Weiden i.d. Opf.	0.036	16074	Saale-Holzland-Kreis	0.063
14521	Erzgebirgskreis	0.008	16061	Eichsfeld	0.058
9184	München	0.005	16070	Ilm-Kreis	0.055
6411	Darmstadt	0.005	3453	Cloppenburg	0.027
			15003	Magdeburg	0.017
			4012	Bremerhaven	0.007

Note: Donor pools corresponds to SCM estimations in Figure S14. Sample weights are chosen to minimize the RMSPE ten days prior to the start of the treatment.

D Single and multiple treatment analyses

D.1 Single treatment analysis in other German cities and regions

In addition to Jena, we estimated treatment effects in Nordhausen (Thuringia, April 14), Rottweil (Baden Württemberg, April 17), Main-Kinzig-Kreis (Hessia, April 20), and Wolfsburg (Lower Saxony, April 20).

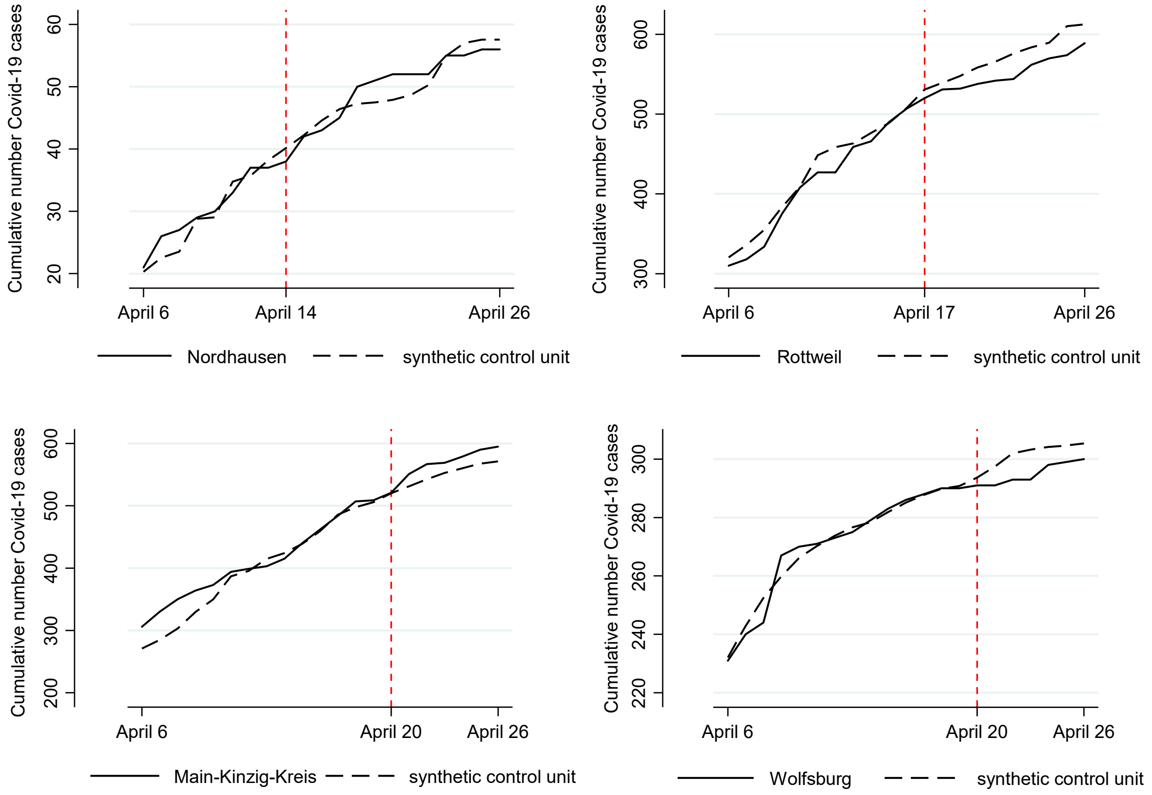

Figure S15: Treatment effects for introduction of face masks in other cities

Notes: Vertical dashed lines (in red color) indicate the introduction of mandatory face masks in each region.

We ignore Braunschweig here as the introduction of face masks became effective only two days in advance of its federal state. Predictor variables are chosen as for overall specification shown in Figure 1. As the figure shows, the result is 2:1:1. Rottweil and Wolfsburg display a positive effect of mandatory mask wearing, just as Jena. The results in Nordhausen are very small or unclear. In the region of Main-Kinzig, it even seems to be the case that masks increased the number of cases relative to the synthetic control group. As all of these regions introduced masks after Jena, the time period available to identify effects is smaller than for Jena. The effects of mandatory face masks could also be underestimated as announcement effects and learning from Jena might have induced individuals to wear masks already before they became mandatory. Finally, the average pre-treatment RMSPE for these four regions (7.150) is larger than for the case of Jena (3.145). For instance, in the case of the region of Main-Kinzig it is more than three times as high (9.719), which indicates a lower pre-treatment fit. The obtained treatment effects should then be interpreted with some care as the pre-treatment estimation error could also translate into the treatment period. In order to minimize the influence of a poor pre-treatment fit for some individual regions, in the main text, we therefore compare the results for Jena with SCM estimates for multiple treated units.

Table S12: SCM weights for control regions in synthetic control groups (other treated NUTS3 regions)

	**Nordhausen**			**Rottweil**	
**ID**	**NUTS 3 region**	**Weight**	**ID**	**NUTS 3 region**	**Weight**
16069	Hildburghausen	0.228	8327	Tuttlingen	0.324
6636	Werra-Meißner-Kreis	0.209	5966	Olpe	0.216
16064	Unstrut-Hainich-Kreis	0.168	8136	Ostalbkreis	0.2
16054	Suhl	0.109	16071	Weimarer Land	0.063
3402	Emden	0.093	14521	Erzgebirgskreis	0.06
12073	Uckermark	0.071	3102	Salzgitter	0.043
12053	Frankfurt (Oder)	0.07	16061	Eichsfeld	0.035
3354	Lüchow-Dannenberg	0.051	9187	Rosenheim	0.031
			9279	Dingolfing-Landau	0.025
			3455	Friesland	0.003
	**Main-Kinzig-Kreis**			**Wolfsburg**	
**ID**	**NUTS 3 region**	**Weight**	**ID**	**NUTS 3 region**	**Weight**
8136	Ostalbkreis	0.193	8212	Karlsruhe	0.357
1062	Stormarn	0.168	8221	Heidelberg	0.189
5966	Olpe	0.113	8211	Baden-Baden	0.158
6433	Groß-Gerau	0.105	10046	St. Wendel	0.128
9473	Coburg	0.092	14511	Chemnitz	0.071
5562	Recklinghausen	0.063	5117	Mülheim an der Ruhr	0.059
7313	Landau in der Pfalz	0.059	5315	Köln	0.028
9171	Altrötting	0.056	15003	Magdeburg	0.007
7338	Rhein-Pfalz-Kreis	0.047	9663	Würzburg	0.004
6437	Odenwaldkreis	0.041			
8236	Enzkreis	0.041			
3159	Göttingen	0.023			

Notes: Donor pools corresponds to SCM estimations in Figure S15. Sample weights are chosen to minimize the RMSPE ten days prior to the start of the treatment.

D.2 Growth rates and summary of single and multiple treatment effects

Jena has 142 registered cases on April 6 compared to an estimated number of 143 cases in the synthetic control group. On April 26 Jena counts 158 cases and the synthetic control group reports 205 (again estimated) cases. The daily growth rate in Jena is denoted by Δx_{Jena} and can be computed from 142 $[1+\Delta x_{Jena}]^{20}$ = 158. The daily growth rate in the control group is denoted by $x_{control}$ and can be computed from 143 $[1+\Delta x_{control}]^{20}$ = 205. Hence, the introduction of the face mask is associated with a decrease in the number of infections of ($\Delta x_{control} - \Delta x_{Jena}$) percentage points per day. Analogously, we also calculate differences in the daily growth rates for our SCM analysis including multiple treated units. The results are summarized in the following table.

Table S13: Summary of treatment effects of face mask introduction in Germany

Difference between treated region(s) and synthetic control group(s)	Single Treatment (Jena)	Multiple treatments (all districts)	Multiple treatments (larger cities)
Absolute change in cumulative number of Covid-19 cases over 20 days	-46.9	-7.0	-28.4
Percentage change in cumulative number of Covid-19 cases over 20 days	-22.9%	-2.6%	-8.9%
Percentage change in newly registered Covid-19 cases over 20 days	-75.6%	-15.7%	-51.2%
Difference in daily growth rates of Covid-19 cases in percentage points	-1.28%	-0.13%	-0.46%
Reduction in daily growth rates of Covid-19 cases (in percent)	70.6%	14.0%	47.3%

All indicators in this table are compiled in an Excel-file available as supporting information on the webpage of the journal.

E A brief survey of research on public health measures against Covid-19

E.1 General overview

Consolidated scientific knowledge on Covid-19 and public health measures taken to fight its epidemic spread, though rapidly evolving, is still limited. Our approach is in line with various studies that have already tried to better understand the effect of public health measures on the spread of Covid-19 (5, 6, 31–37). However, these earlier studies all take an aggregate approach in the sense that they look at implementation dates for a certain measure and search for subsequent changes in the national incidence. There are some prior analyses that take a regional focus (7) but no attention is paid to the effect of policy measures.[29]

There are also many cross-country analyses, both in a structural SIR (susceptible, infectious and removed) sense (39) and with an econometric focus on forecasting the future development of the Covid-19 pandemic (40). Others draw parallels between earlier pandemics and Covid-19 (41). These studies do not explicitly take public health measures into account. Some studies discuss potential effects of public health measures and survey general findings (42–44) but do not provide direct statistical evidence on specific measures.

The synthetic control method (SCM) has been applied by (16) to estimate the effect of the shelter-in-place order for California, USA, in the development of Covid-19. The authors find *inter alia* that around 1600 deaths from Covid-19 were avoided by this measure during the first four weeks. (45) use SCM to study the case of Sweden as one of the few countries without a lock down. The results indicate that the infection dynamics in the synthetic control group (constructed from a donor pool of other European countries) does not systematically differ from

[29] In a short note, (38) apply panel methods based on time dummies to understand the relative importance of various public health measures. They employ data at the federal state level and not at the regional level. As a detailed model description is not available, an appreciation of results is difficult at this point.

the actual dynamics in Sweden. Based on Google mobility data, the authors further find that Swedes adjusted their activities in similar ways as in the synthetic control group even without a mandated lock down.

E.2 Evidence for face masks

At present, more and more clinical evidence is being presented. While final conclusive results have not been reached (see ref. 46 for a review), research shows that face masks catch infectious particles that occur when speaking, coughing, or sneezing. This reduces the risk of infecting another person (47, 48). The effects of face masks have been systematically surveyed by (49) and (50). (50) mainly present evidence on the effect of face masks during non-Covid epidemics (influenza and SARS). (51) reports that they "*did not find any studies that investigated the effectiveness of face mask use in limiting the spread of COVID-19 among those who are not medically diagnosed with COVID-19 to support current public health recommendations*".

In addition to medical aspects (like transmission characteristics of Covid-19 and filtering capabilities of masks), (49) survey evidence on mask efficiency and on the effect of a population. They first stress that "*no randomized control trials on the use of masks <…> has been published*". The study which is "*the most relevant paper*" for (49) is one that analyzed *"exhaled breath and coughs of children and adults with acute respiratory illness*" (52, p. 676), i.e. used a clinical setting. Concerning the effect of masks on community transmissions, the survey needs to rely on pre-Covid-19 studies.

Only very recently, first non-clinical observational studies on the effectiveness of face masks have been published. The work that is most closely related to our approach is (53), who estimate the effects of public health measures on the spread of Covid-19 in the three pandemic epicenters Wuhan, Italy, and New York City over the period January 23 to May 9, 2020. The authors find sizable effects for the introduction of face masks, indicating that this public health measure alone reduced the number of infections by over 78,000 in Italy from April 6 to May 9 and by over 66,000 in New York City from April 17 to May 9.

The authors adopt an empirical identification strategy that utilizes the successive implementation of individual public health measures and estimate linear time trends for the period before the introduction of face masks in Italy and New York City. The difference between these trends and actual Covid-19 cases is interpreted as the mitigating effect of mandated face covering. Although the authors argue that their trend projections are reasonable considering the excellent linear correlation for the data prior to the onset of mandated face covering, a limitation is that their study does not employ a strict control group approach and conducts inference on in a "before-after" comparison, which may not suffice to rule out all confounding factors.[30]

(54) use household data for 335 families in Beijing with at least one confirmed Covid-19 case to study factors that influence disease transmission within families. The authors track the rate of secondary transmissions over the two weeks of follow-up from onset of the primary case within the family. Findings suggest that transmission was significantly reduced by frequent use of chlorine or ethanol-based disinfectant in households and family members (including the primary case) wearing a face mask at home before the primary case developed the illness. The authors motivate their findings for wearing face masks early one by the fact that the viral load is highest

[30] Although the authors compare their findings for Italy and New York City with global Covid-19 trends in the world and in the United States, the lack of a suitable comparison groups cannot rule out that some unobserved factors in Italy and New York City other than the introduction of face masks drove the estimated trend reversal.

in the 2 days before symptom onset and on the first day of symptoms, and up to 44% of transmission is during the pre-symptomatic period.

Finally, (55) use a simulation study to assess the role of face masks on the epidemic spread with or without other public health measures being simultaneously in place. Their findings indicate that face masks can effectively mitigate the epidemic spread if they are used by the public all the time (not just from when symptoms first appear). The simulated effects are the greatest when the adoption rate of wearing face masks in the public is 100 percent and when it is combined with an early lock-down situation. When interpreting their simulation results, the authors stress that accurate experimental evidence for potential control interventions would be needed to fully evaluate the effect of face masks.

E.3 The economic costs of public health measures

We provide a short overview of studies that quantify economic costs of public health measures and a first rough estimate of the cost of face masks. For the case of school and child-care center closures, (56) estimate that 8.4 percent of total working hours will be lost, corresponding to 11.7 million employed persons in short-time-work. Beyond this short-term effect, intergenerational mobility and gender equality in the workplace is likely negatively affected. The macroeconomic impact of this public health measure is considered large.

Substantial GDP impacts of closures of stores, restaurants and other business outlets are found by (57) under various degrees of easing restrictions. Costs of face masks are neglected when assessing overall economic consequences of all measures. Economic effects of several interventions are examined by (58) for New Zealand. While a moderate decline in GDP of 0.7 percent is estimated for the ban of mass gatherings and closure of public venues, restricting domestic travel may induce a large reduction of up to 6 percent. Economic effects of social-distances policies are investigated by (59) and (60). The advantageousness of testing from the macroeconomic point of view is highlighted by (61) and (62). Finally, several papers examine macroeconomic effects of a general lockdown (cf. ref. 63–65).

We conduct a simple back-of-the-envelope calculation in order to understand the cost burden from wearing face masks for private household. Each person may need two face masks a day. In September 2020, two simple single use masks certainly cost less than 1 € in Germany. This translates to expenditures of less than 30 € per month. According to (66), the average monthly disposable income per one-person household was 2112€ in 2017 in Germany. Monthly per capita expenditures for face masks should therefore account for less than 1.4% of the average disposable income. We can do similar calculations for larger households. They have to spend a larger share of their disposable income.[31] Of course, the monthly expenditures may be reduced if fabric masks are used. Washing and using them again is possible. We conclude that in relation to other public health measures and their consequences (among which part-time work or unemployment), the cost burden of face masks may be low for private households.

[31] Two-person households had an average monthly disposable income of 3919 € in 2017 (66). They may need four masks per day, which translates to expenditures of less than 1.5% of the average disposable income for simple face masks. Expenditures for a four person household (average monthly disposable income of 5483 € in 2017, 8 masks may be needed per day) should account for less than 2.5% of the average disposable income.

F Difference-in-difference estimates for timing of treatment effects

One difficulty in the empirical identification of treatment effects of face masks relates to the fact that Jena has introduced several public health measures to fight the local spread of Covid-19 in rapid procession over time. An overview is given in Figure S2 above. We emphasized that some of these measures in Jena (light colors) deviate from their general introduction at the federal state level (dark colors). These anticipated measures may be taken as a signal for the severity of the pandemic and, accordingly, may have induced behavioral changes of the local population even before face masks became compulsory. To test for the strength of such dynamic treatment effects over time, we complement our SCM approach by conducting incremental difference-in-difference (IDiD) estimation (67; see ref. 68 for a general discussion of the use of difference-in-difference estimation to identify causal effects of Covid-19 policies).

In order to test for the presence of anticipation effects stemming from (unobserved) public health measures taken prior to the introduction of face masks, we define a baseline treatment dummy, which takes a value of one for Jena from March 14 onwards and is zero before that day. This captures the start of public health measures taken in Jena (compare with Figure S2). We include this treatment dummy in a fixed effect (FE) regression model, which uses the (log-transformed) cumulative number of Covid-19 cases as outcome variable. Starting from this baseline treatment specification, we run a series of regressions where each adds a second treatment dummy to the model. The latter takes a value of one for Jena from day *m* onwards and is zero before that day. We allow *m* to vary between March 15 and April 25. The sample as a whole ends on May 6.

Hence, the main idea of the proposed IDiD approach is to see whether we observe a general treatment effect with the start of public health measures on March 14. On top, we can identify additional effects, which relate to (unobserved) public measures introduced during the time interval. Again, as outlined in SI Appendix, Section A.3, for the correct interpretation of the obtained results, we need to account for the time lag resulting from an incubation period and a reporting lag to health authorities.

Formally the *m*-th equation for a total of *m*=(1,…,*M*) regressions takes the following form,

$$covid_{i,t} = \beta \times \Delta covid_{i,t-1} + \gamma \times base_{i,t} + \delta_m \times add_{i,t}^m + D_{Weekday} + \mu_i + \Psi_{k(t)} + e_{i,t},$$

where $covid_{i,t}$ denotes the (log-transformed) cumulative number of registered Covid-19 cases in municipal district *i* at day *t* with $i = 1, \dots, N$ and $t = 1, \dots, T$. $\Delta covid_{i,t}$ is the number of newly registered Covid-19 cases at day *t-1*. $base_{i,t}$ refers to the baseline treatment dummy and $add_{i,t}^m$ is the additional treatment dummy from day *m* onwards. Further, μ_i are region-fixed effects at the level of municipal districts, $D_{Weekday}$ is a set of binary dummies for the different days of the week and $\Psi_{k(t)}$ are time-fixed effects for each with *k=1,…,K* calendar week in the sample period. $e_{i,t}$ denotes the model's *i.i.d.* error term. We are mostly interested in estimating γ and δ_m, which sum up to the overall treatment effect of public health measures in Jena taken from March 14 onwards.

We estimate the FE-based IDiD model by means of weighted least square (WLS), where weights are generated from a first step Probit regression with $base_{i,t}$ as the outcome variable. We estimate the Probit model as a cross-sectional specification for March 14 and include values of newly registered Covid-19 cases before March 14 as well as the set of structural regional characteristics (shown in Table S4 in SI Appendix, Section C.1) as regressors. Hence, in analogy to

our SCM approach, the main idea for this two-step approach is to give those control regions a larger sample weight that have similar characteristics to Jena before the baseline treatment starts (68). This may mitigate the problem of DiD estimation to result in a poor performance potentially linked to the problem of heteroscedasticity if there are very few (or even only one) treatment group (see ref. 69 for a general discussion of inference in DiD models with few treated units). The resulting two-step estimator is known as conditional difference-in-difference estimator (70). Estimated effects are shown in Figure S16.

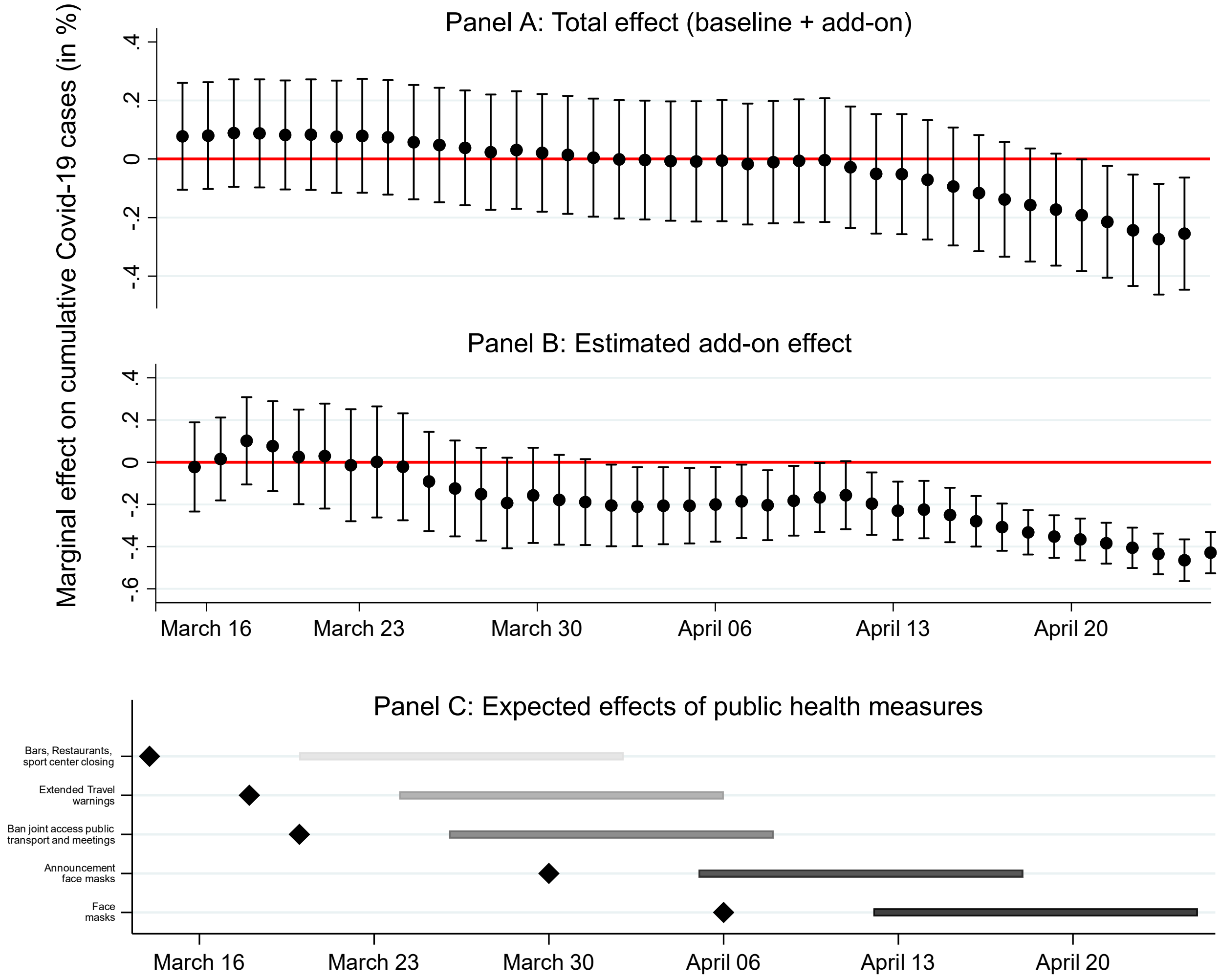

Figure S16: Estimated effects from incremental difference-in-difference (IDiD) model

Notes: We calculate point estimates and standard errors for the total treatment effect $(\gamma + \delta_m)$ on the basis of the Delta method. In Panel A and Panel B solid lines indicate 95% confidence intervals for reported point estimates. Standard errors in the FE-model are clustered at the municipal district level. In Panel C markers indicate the start of a specific public health measures; bars indicate the range of expected effects taking an incubation period and reporting delay into account.

The figure shows the second-step IDiD regression results for the total treatment effect $(\gamma + \delta_m)$ in Panel A and the add-on treatment effect (δ_m) in Panel B. Panel C shows the expected timing of effects for different public health measures if we consider a total delay *D* of 19 days for the incubation period and an associated reporting lag. As shown in Table S3 in SI Appendix, Section A.3, this covers more than 90% of cases associated with a specific date (i.e. timing of

public health measure). Estimations are based on a sample of 20 regions (19 controls with positive sample weights plus Jena) during the sample period January 28 until May 6 (with a total number of 1,980 observations).

We find that the total treatment effect for public health measures in Jena relative to the control group only becomes significant roughly two weeks after the introduction of face masks on April 6. This strongly overlaps with expected effects stemming from the announcement and introduction of compulsory face masks in Jena (as shown in Panel C). In terms of the magnitude of the effect, we find a reduction in the cumulative number of Covid-19 cases by roughly 20%. Both findings are in line with our baseline SCM approach.

While Panel B of Figure S16 shows that we find marginally significant add-on effects from early April on, their magnitude is not sufficient to translate into a significant reduction in the number of Covid-19 cases vis-à-vis the set of control regions. Only from April 13 onwards, thus roughly one week after the introduction of face masks, the add-on treatment effect becomes gradually stronger in magnitude and statistically significant. If we resort to the total delay *D* as estimated in SI Appendix, Section A.3, this result further supports our SCM findings that the relative reduction in the cumulative Covid-19 cases is mainly attributable to the announcement/introduction of face masks.

Table S14: Control regions included in the IDiD estimation

**ID**	**NUTS3 region**
2000	Hamburg
3101	Braunschweig
3102	Salzgitter
3103	Wolfsburg
5315	Köln
5515	Münster
6411	Darmstadt
6412	Frankfurt am Main
7315	Mainz
8111	Stuttgart
8212	Karlsruhe
8221	Heidelberg
8222	Mannheim
9161	Ingolstadt
9562	Erlangen
14511	Chemnitz
14612	Dresden
14713	Leipzig
16051	Erfurt

Notes: Selection of regions is based on Probit regression with the baseline treatment dummy in Jena on March 14 as outcome variable (see text in this SI Appendix section for details). In the FE-specification reported in Figure S16, we have set sample weights for selected control regions equal to one; alternative specifications with changing weights deliver very similar results and are not explicitly reported here (regression outputs are in the zip-file on the Journal's web page).

References for the SI Appendix

1. N. M. Linton, T. Kobayashi, Y. Yang, K. Hayashi, A. R. Akhmetzhanov, S.-M. Jung, B. Yuan, R. Kinoshita, H. Nishiura, Incubation Period and Other Epidemiological Characteristics of 2019 Novel Coronavirus Infections with Right Truncation: A Statistical Analysis of Publicly Available Case Data. *Journal of clinical medicine* **9 (**2020).
2. S. A. Lauer, K. H. Grantz, Q. Bi, F. K. Jones, Q. Zheng, H. R. Meredith, A. S. Azman, N. G. Reich, J. Lessler, The Incubation Period of Coronavirus Disease 2019 (COVID-19) From Publicly Reported Confirmed Cases: Estimation and Application. *Annals of internal medicine* **172**, 577–582 (2020).
3. Robert Koch Institute, Covid-19 Infektionen, General Website (NPGEO Corona Hub). https://npgeo-corona-npgeo-de.hub.arcgis.com/, accessed on June 4, 2020.
4. U. Buchholz, S. Buda, K. Prahm, Abrupter Rückgang der Raten an Atemwegserkrankungen in der deutschen Bevölkerung. *Epidemiologisches Bulletin*. 10.25646/6636.2, 7–9 (2020).
5. Donsimoni, J. R., R. Glawion, B. Plachter & K. Wälde, Projecting the Spread of COVID-19 for Germany. *German Economic Review* **21**, 181–216 (2020).
6. Donsimoni, J. R., R. Glawion, B. Plachter, K. Wälde & C. Weiser, Should Contact Bans Be Lifted in Germany? A Quantitative Prediction of Its Effects. *CESifo Economic Studies* **66**, 115–133 (2020).
7. S. Khailaie, T. Mitra, A. Bandyopadhyay, M. Schips, P. Mascheroni, P. Vanella, B. Lange, S. Binder, M. Meyer-Hermann, Estimate of the development of the epidemic reproduction number Rt from Coronavirus SARS-CoV-2 case data and implications for political measures based on prognostics. Available at http://dx.doi.org/10.1101/2020.04.04.20053637 (deposited 2020).
8. T. Harko, F. S. Lobo, M. K. Mak, Exact analytical solutions of the Susceptible-Infected-Recovered (SIR) epidemic model and of the SIR model with equal death and birth rates. *Applied Mathematics and Computation* **236**, 184–194 (2014).
9. A. A. Toda, Susceptible-infected-recovered (SIR) dynamics of Covid-19 and economic impact. Covid Economics. 1, 43–63. Available at: https://cepr.org/sites/default/files/news/CovidEcon1%20final.pdf.
10. A. Abadie, Using Synthetic Controls: Feasibility, Data Requirements, and Methodological Aspects. *[Forthcoming in] Journal of Economic Literature*. 10.1257/jel.20191450.
11. A. Abadie, J. Gardeazabal, The Economic Costs of Conflict: A Case Study of the Basque Country. *American Economic Review* **93**, 113–132 (2003).
12. S. O. Becker, S. Heblich, D. Sturm, The impact of public employment: Evidence from Bonn. IZA Discussion Paper No. 11255. https://www.iza.org/publications/dp/11255.
13. S. Athey, G. W. Imbens, The State of Applied Econometrics: Causality and Policy Evaluation. *Journal of Economic Perspectives* **31**, 3–32 (2017).
14. N. Kreif, R. Grieve, D. Hangartner, A. J. Turner, S. Nikolova, M. Sutton, Examination of the Synthetic Control Method for Evaluating Health Policies with Multiple Treated Units. *Health economics* **25**, 1514–1528 (2016).

15. H. Pieters, D. Curzi, A. Olper, J. Swinnen, Effect of democratic reforms on child mortality: a synthetic control analysis. *The Lancet Global Health* **4**, e627-e632 (2016).

16. A. Friedson, D. McNichols, J.J. Sabia, D. Dave, Did California's Shelter-In-Place Order Work? Early Coronavirus-Related Public Health Effects. IZA Discussion Paper no. 13160. Available at: https://www.iza.org/publications/dp/13160.

17. A. Abadie, A. Diamond, J. Hainmueller, Synthetic Control Methods for Comparative Case Studies: Estimating the Effect of California's Tobacco Control Program. *Journal of the American Statistical Association* **105**, 493–505 (2010).

18. E. Cavallo, S. Galiani, I. Noy, J. Pantano, Catastrophic Natural Disasters and Economic Growth. *Review of Economics and Statistics* **95**, 1549–1561 (2013).

19. N. F. Campos, F. Coricelli, L. Moretti, Institutional integration and economic growth in Europe. *Journal of Monetary Economics* **103**, 88–104 (2019).

20. K. Wälde, Corona-Blog, Einschätzung vom Freitag, 20. März 2020: https://www.macro.economics.uni-mainz.de/corona-blog/, accessed June 4, 2020.

21. P. Eliason, B. Lutz, Can fiscal rules constrain the size of government? An analysis of the "crown jewel" of tax and expenditure limitations. *Journal of public economics* **166**, 115–144 (2018).

22. L. Hu, R. Kaestner, B. Mazumder, S. Miller, A. Wong, The Effect of the Affordable Care Act Medicaid Expansions on Financial Wellbeing. *Journal of public economics* **163**, 99–112 (2018).

23. S. Galiani, B. Quistorff, The Synth_Runner Package: Utilities to Automate Synthetic Control Estimation Using Synth. *The Stata Journal* **17**, 834–849 (2017).

24. Alberto Abadie & Alexis Diamond & Jens Hainmueller, SYNTH: Stata module to implement Synthetic Control Methods for Comparative Case Studies: Boston College Department of Economics, revised 09 May 2020. *Statistical Software Components (*2011).

25. S. Firpo, V. Possebom, Synthetic Control Method: Inference, Sensitivity Analysis and Confidence Sets. *Journal of Causal Inference* **6 (**2018).

26. INKAR, Indikatoren und Karten zur Raum- und Stadtentwicklung: Bundesinstitut für Bau-, Stadt- und Raumforschung (Federal Institute for Research on Building, Urban Affairs and Spatial Development). https://www.inkar.de/, accessed on June 4, 2020.

27. Google LLC, Google Trends. https://trends.google.de/trends/, accessed June 4, 2020 (deposited 2020).

28. Google LLC, COVID-19 Community Mobility reports: https://www.google.com/covid19/mobility/, accessed June 4, 2020 (deposited 2020).

29. Google LLC, COVID-19 Community Mobility Reports, Mobility Report CSV Documentation: https://www.google.com/covid19/mobility/data_documentation.html?hl=en, accessed on June 4, 2020.

30. Thüringer Allgemeine, Infektionsherde ließen Corona-Zahlen im Kreis Gotha steigen: https://www.thueringer-allgemeine.de/regionen/gotha/infektionsherde-liessen-corona-

zahlen-im-kreis-gotha-steigen-id229070201.html, dated 8 May 2020, accessed September 9, 2020 (deposited 2020).

31. W. Bock, B. Adamik, M. Bawiec, V. Bezborodov, M. Bodych, J. P. Burgard, T. Goetz, T. Krueger, A. Migalska, B. Pabjan, T. Ozanski, E. Rafajlowicz, W. Rafajlowicz, E. Skubalska-Rafajlowicz, S. Ryfczynska, E. Szczurek, P. Szymanski, Mitigation and herd immunity strategy for COVID-19 is likely to fail. Available at http://dx.doi.org/10.1101/2020.03.25.20043109 (deposited 2020).

32. M. V. Barbarossa, J. Fuhrmann, J. H. Meinke, S. Krieg, H. V. Varma, N. Castelletti, T. Lippert, The impact of current and future control measures on the spread of COVID-19 in Germany. Available at http://dx.doi.org/10.1101/2020.04.18.20069955 (deposited 2020).

33. G. Bonaccorsi, F. Pierri, M. Cinelli, A. Flori, A. Galeazzi, F. Porcelli, A. L. Schmidt, C. M. Valensise, A. Scala, W. Quattrociocchi, F. Pammolli, Economic and social consequences of human mobility restrictions under COVID-19. *Proceedings of the National Academy of Sciences of the United States of America* **117**, 15530–15535 (2020).

34. N. G. Davies, A. J. Kucharski, R. M. Eggo, A. Gimma, W. J. Edmunds, T. Jombart, K. O'Reilly, A. Endo, J. Hellewell, E. S. Nightingale, B. J. Quilty, C. I. Jarvis, T. W. Russell, P. Klepac, N. I. Bosse, S. Funk, S. Abbott, G. F. Medley, H. Gibbs, C. A. B. Pearson, S. Flasche, M. Jit, S. Clifford, K. Prem, C. Diamond, J. Emery, A. K. Deol, S. R. Procter, K. van Zandvoort, Y. F. Sun, J. D. Munday, A. Rosello, M. Auzenbergs, G. Knight, R. M. G. J. Houben, Y. Liu, Effects of non-pharmaceutical interventions on COVID-19 cases, deaths, and demand for hospital services in the UK: a modelling study. *The Lancet Public Health* **5**, e375-e385 (2020).

35. J. Dehning, J. Zierenberg, F. P. Spitzner, M. Wibral, J. P. Neto, M. Wilczek, V. Priesemann, Inferring change points in the spread of COVID-19 reveals the effectiveness of interventions. *Science*. 10.1126/science.abb9789 (2020).

36. C. Gros, R. Valenti, L. Schneider, K. Valenti, D. Gros, Containment efficiency and control strategies for the Corona pandemic costs. http://arxiv.org/pdf/2004.00493v2 (deposited 2020).

37. T. Hartl, K. Wälde, E. Weber, Measuring the impact of the German public shutdown on the spread of Covid-19. Covid Economics. 1, 25–32. Available at: https://cepr.org/sites/default/files/news/CovidEcon1%20final.pdf.

38. T. Hartl, E. Weber, Welche Maßnahmen brachten Corona unter Kontrolle? Available at: https://www.oekonomenstimme.org/artikel/2020/05/welche-massnahmen-brachten-corona-unter-kontrolle/ (deposited 2020).

39. Chen, X. & Z. Qiu, Scenario analysis of non-pharmaceutical interventions on global Covid-19 transmissions. Covid Economics. 7, 46–67. Available at: https://cepr.org/sites/default/files/news/CovidEconomics7.pdf.

40. A. Ritschl, Visualising and forecasting Covid-19. Covid Economics. 5, 79–105. Available at: https://cepr.org/sites/default/files/news/CovidEconomics5.pdf.

41. Barro, Robert J. and Ursua, José F and Weng, Joanna, The Coronavirus and the Great Influenza Epidemic - Lessons from the 'Spanish Flu' for the Coronavirus's Potential Effects on Mortality and Economic Activity. CESifo Working Paper No. 8166. Available at: https://www.cesifo.org/node/53868 (deposited 2020).

42. A. Wilder-Smith, C. J. Chiew, V. J. Lee, Can we contain the COVID-19 outbreak with the same measures as for SARS? *The Lancet. Infectious Diseases*. 10.1016/S1473-3099(20)30129-8 (2020).

43. R. M. Anderson, H. Heesterbeek, D. Klinkenberg, T. D. Hollingsworth, How will country-based mitigation measures influence the course of the COVID-19 epidemic? *The Lancet* **395**, 931–934 (2020).

44. N. Ferguson, D. Laydon, G. Nedjati Gilani, N. Imai, K. Ainslie, M. Baguelin, S. Bhatia, A. Boonyasiri, Z. Cucunuba Perez, G. Cuomo-Dannenburg, A. Dighe, I. Dorigatti, H. Fu, K. Gaythorpe, W. Green, A. Hamlet, W. Hinsley, L. Okell, S. van Elsland, H. Thompson, R. Verity, E. Volz, H. Wang, Y. Wang, P. Walker, P. Winskill, C. Whittaker, C. Donnelly, S. Riley, A. Ghani, Report 9: Impact of non-pharmaceutical interventions (NPIs) to reduce COVID19 mortality and healthcare demand. Medical Research Council (MRC). Available at http://dx.doi.org/10.25561/77482 (deposited 2020).

45. Born, B., A. Dietrich & G. Müller, Do lockdowns work? A counterfactual for Sweden.: CEPR Discussion Paper No. 14744. Available at: https://cepr.org/active/publications/discussion_papers/dp.php?dpno=14744 (deposited 2020).

46. G. Seminara, B. Carli, G. Forni, S. Fuzzi, A. Mazzino, A. Rinaldo, Biological fluid dynamics of airborne COVID-19 infection. *Rendiconti Lincei. Scienze fisiche e naturali*. 10.1007/s12210-020-00938-2, 1–33 (2020).

47. D. K. Chu, E. A. Akl, S. Duda, K. Solo, S. Yaacoub, H. J. Schünemann, A. El-harakeh, A. Bognanni, T. Lotfi, M. Loeb, A. Hajizadeh, A. Bak, A. Izcovich, C. A. Cuello-Garcia, C. Chen, D. J. Harris, E. Borowiack, F. Chamseddine, F. Schünemann, G. P. Morgano, G. E. U. Muti Schünemann, G. Chen, H. Zhao, I. Neumann, J. Chan, J. Khabsa, L. Hneiny, L. Harrison, M. Smith, N. Rizk, P. Giorgi Rossi, P. AbiHanna, R. El-khoury, R. Stalteri, T. Baldeh, T. Piggott, Y. Zhang, Z. Saad, A. Khamis, M. Reinap, Physical distancing, face masks, and eye protection to prevent person-to-person transmission of SARS-CoV-2 and COVID-19: a systematic review and meta-analysis. *The Lancet* **395**, 1973–1987 (2020).

48. K. A. Prather, C. C. Wang, R. T. Schooley, Reducing transmission of SARS-CoV-2. *Science*. 10.1126/science.abc6197 (2020).

49. J. Howard, A. Huang, Z. Li, Z. Tufekci, V. Zdimal, H.-M. van der Westhuizen, A. von Delft, A. Price, L. Fridman, L.-H. Tang, V. Tang, G. L. Watson, C. E. Bax, R. Shaikh, F. Questier, D. Hernandez, L. F. Chu, C. M. Ramirez, A. W. Rimoin, Face Masks Against COVID-19: An Evidence Review. Preprints 2020040203. Available at http://dx.doi.org/10.20944/preprints202004.0203.v1 (deposited 2020).

50. T. Greenhalgh, M. B. Schmid, T. Czypionka, D. Bassler, L. Gruer, Face masks for the public during the covid-19 crisis. *BMJ (Clinical research ed.)* **369**, m1435 (2020).

51. K. M. Marasinghe, Concerns around public health recommendations on face mask use among individuals who are not medically diagnosed with COVID-19 supported by a systematic review search for evidence. Available at http://dx.doi.org/10.21203/rs.3.rs-16701/v3 (deposited 2020).

52. N. H. L. Leung, D. K. W. Chu, E. Y. C. Shiu, K.-H. Chan, J. J. McDevitt, B. J. P. Hau, H.-L. Yen, Y. Li, D. K. M. Ip, J. S. M. Peiris, W.-H. Seto, G. M. Leung, D. K. Milton, B. J. Cowling,

Respiratory virus shedding in exhaled breath and efficacy of face masks. *Nature medicine* **26**, 676–680 (2020).

53. R. Zhang, Y. Li, A. L. Zhang, Y. Wang, M. J. Molina, Identifying airborne transmission as the dominant route for the spread of COVID-19. *Proceedings of the National Academy of Sciences of the United States of America* **117**, 14857–14863 (2020).

54. Y. Wang, H. Tian, L. Zhang, M. Zhang, D. Guo, W. Wu, X. Zhang, G. L. Kan, L. Jia, D. Huo, B. Liu, X. Wang, Y. Sun, Q. Wang, P. Yang, C. R. MacIntyre, Reduction of secondary transmission of SARS-CoV-2 in households by face mask use, disinfection and social distancing: a cohort study in Beijing, China. *BMJ global health* **5 (**2020).

55. R. O. J. H. Stutt, R. Retkute, M. Bradley, C. A. Gilligan, J. Colvin, A modelling framework to assess the likely effectiveness of facemasks in combination with 'lock-down' in managing the COVID-19 pandemic. *Proc. R. Soc. A.* **476**, 20200376 (2020).

56. Fuchs-Schündeln, Nicola and Kuhn, Moritz and Tertilt, Michèle, The Short-Run Macro Implications of School and Child-Care Closures. IZA Discussion Paper No. 13353, Available at: https://www.iza.org/publications/dp/13353 (deposited 2020).

57. A. Basurto, H. Dawid, P. Harting, J. Hepp, D. Kohlweyer, Economic and epidemic implications of virus containment policies: insights from agent-based simulations. Universität Bielefeld Working Papers in Economics and Management; 05-2020. Available at http://dx.doi.org/10.4119/unibi/2944282 (deposited 2020).

58. Stannard, T. and G. Steven and C. McDonald, Economic impacts of COVID-19 containment measures. Reserve Bank of New Zealand Analytical Notes AN2020/04, Reserve Bank of New Zealand. https://www.rbnz.govt.nz/-/media/reservebank/files/publications/analytical%20notes/2020/an2020-04.pdf (deposited 2020).

59. A. Strong, J. Welburn, An Estimation of the Economic Costs of Social-Distancing Policies. Santa Monica, CA: RAND Corporation, (2020). Available at http://dx.doi.org/10.7249/RRA173-1 (deposited 2020).

60. J. Fernández-Villaverde, C. Jones, Estimating and Simulating a SIRD Model of COVID-19 for Many Countries, States, and Cities. NBER Working Paper #27128. Available at http://dx.doi.org/10.3386/w27128 (deposited 2020).

61. Brotherhood, Luiz and Kircher, Philipp and Santos, Cezar and Tertilt, Michèle, An Economic Model of the Covid-19 Epidemic: The Importance of Testing and Age-Specific Policies. IZA Discussion Paper No. 13265. Available at: https://www.iza.org/publications/dp/13265 (deposited 2020).

62. M. Eichenbaum, S. Rebelo, M. Trabandt, The Macroeconomics of Testing and Quarantining. NBER Working Paper #27104. Available at http://dx.doi.org/10.3386/w27104 (deposited 2020).

63. D. Acemoglu, V. Chernozhukov, I. Werning, M. Whinston, Optimal Targeted Lockdowns in a Multi-Group SIR Model. NBER Working Paper 27102, National Bureau of Economic Research. Available at http://dx.doi.org/10.3386/w27102 (deposited 2020).

64. M. Eichenbaum, S. Rebelo, M. Trabandt, Epidemics in the Neoclassical and New Keynesian Models. NBER Working Paper #27430. Available at http://dx.doi.org/10.3386/w27430 (deposited 2020).

65. M. Eichenbaum, S. Rebelo, M. Trabandt, The Macroeconomics of Epidemics. NBER Working Paper #26882. Available at http://dx.doi.org/10.3386/w26882 (deposited 2020).

66. Statistisches Bundesamt, Table 63121-0001, Einkommen und Einnahmen sowie Ausgaben privater Haushalte (Laufende Wirtschaftsrechnungen): Deutschland, Jahre, Haushaltsgröße, Laufende Wirtschaftsrechnungen: Haushaltsbuch. Available at:. https://www-genesis.destatis.de/genesis/online, accessed on September 30, 2020.

67. P. Dolton, C. R. Bondibene, J. Wadsworth, The UK National Minimum Wage in Retrospect. *Fiscal Studies* **31**, 509–534 (2010).

68. A. Goodman-Bacon, J. Marcus, Using Difference-in-Differences to Identify Causal Effects of COVID-19 Policies. *Survey Research Methods*. 10.18148/srm/2020.v14i2.7723, 153–158 (2020).

69. B. Ferman, C. Pinto, Inference in Differences-in-Differences with Few Treated Groups and Heteroskedasticity. *Review of Economics and Statistics* **101**, 452–467 (2019).

70. J. Heckman, H. Ichimura, J. Smith, P. Todd, Characterizing Selection Bias Using Experimental Data. *Econometrica* **66**, 1017 (1998).

Content of SI Appendix

A Timing of public health measures and visibility in data 2
A.1 Timing of the introduction of mandatory face masks 2
A.2 The timing of other public health measures 3
A.3 When are effects of public health measures visible in the data? 5
A.4 Visibility in data II – Conceptual background 8
B Synthetic control method: Design, implementation and inference 9
C Data description and additional SCM estimation results for Jena 13
C.1 Summary statistics for outcome and predictor variables 13
C.2 Trajectories of cumulative Covid-19 cases and box plots for predictor variables 14
C.3 Control regions, SCM weights and pre-treatment predictor balance 16
C.4 SCM results by age groups 17
C.5 Effects on cumulative number of infections per 100,000 inhabitants 19
C.6 Announcement and mobility 20
C.6.1 Google trends and announcement effects 20
C.6.2 Mobility trends across German federal states 20
C.7 Cross validation and additional placebo-in-time test 22
C.8 Changes in donor pool for synthetic Jena 23
C.9 Place-in-space tests for other major cities in Thuringia 25
D Single and multiple treatment analyses 26
D.1 Single treatment analysis in other German cities and regions 26
D.2 Growth rates and summary of single and multiple treatment effects 28
E A brief survey of research on public health measures against Covid-19 29
E.1 General overview 29
E.2 Evidence for face masks 30
E.3 The economic costs of public health measures 31
F Difference-in-difference estimates for timing of treatment effects 32
References for the SI Appendix 35
